# Supplementary material for: In vitro antibacterial activity of dinuclear thiolato-bridged ruthenium(II)-arene compounds
Source: Microbiol Spectr. 2023 Oct 10;11(6):e00954-23. doi: 10.1128/spectrum.00954-23 (PMC10714934; doi:10.1128/spectrum.00954-23)
Supplement: Supplemental material — Figures, tables, and spectra. [file spectrum.00954-23-s0001.docx]

***Supporting Information***

**Characterization of the *In Vitro* Activity of Dinuclear Trithiolato-Bridged Ruthenium(II)-Arene Against Bacteria**

Quentin Bugnon^1,2^, Camilo Melendez^2^, Oksana Desiatkina^2^, Louis Fayolle de Chaptes^2^, Isabelle Holzer^2^, Emilia Păunescu^2^, Markus Hilty^1,*^ and Julien Furrer^2,*^

^1^Institute for Infectious Diseases, University of Bern, Friedbühlstrasse 51, 3001, Bern, Switzerland.

^2^Department of Chemistry, Biochemistry and Pharmaceuticals Sciences, University of Bern, Freiestrasse 3, 3012, Bern, Switzerland.

**Table of contents**

|  |  | **Page.** |
| --- | --- | --- |
| **Experimental part – Chemistry** |  | S3 |
| 1. **General** |  | S3 |
| 1. **Synthesis of compounds 17-19** |  | S4 |
| - 1. **Synthesis of ligands 17a-19a** |  | S4 |
| - 1. **Synthesis of diruthenium complexes 17-19** |  | S6 |
| 1. **Synthesis of compounds 20-22** |  | S9 |
| - 1. **Synthesis of ligands and intermediates** |  | S8 |
| - 1. **Synthesis of diruthenium complexes 20, 21 and 22** |  | S12 |
| 1. **NMR spectra of compounds 17a, 17, 18, 19, 22a, 20, 21, 22 and 23** |  | S15 |

**List of figures**

|  |  | **Page.** | |
| --- | --- | --- | --- |
| **Scheme S1.** Synthesis of thiol ligands **17a-19a**.  **Scheme S2.** Synthesis of thiol ligands **23** and **22a** |  | S4  S9 | |
|  |  |  | |
| **Figure S1a.** ^1^H NMR spectrum of diphenyl-(2-mercaptophenyl)phosphonate (**17a**) in CD_2_Cl_2_. |  | S15 | |
| **Figure S1b.** ^13^C NMR spectrum of diphenyl-(2-mercaptophenyl)phosphonate (**17a**) in CD_2_Cl_2_. |  | S15 | |
| **Figure S1c.** ^31^P NMR spectrum of diphenyl-(2-mercaptophenyl)phosphonate (**17a**) in CD_2_Cl_2_ |  | S16 | |
| **Figure S2a.** ^1^H NMR spectrum of [(*η*^6^-*p*-MeC_6_H_4_*^i^*Pr)_2_Ru_2_(*μ*_2_-SCH_2_C_6_H_4_-*p*-*^t^*Bu)_2_(*μ*_2_-SC_6_H_4_-*o*-P(O)(OPh)_2_]Cl (**17**) in CDCl_3_. |  | S16 | |
| **Figure S2b.** ^13^C NMR spectrum of [(*η*^6^-*p*-MeC_6_H_4_*^i^*Pr)_2_Ru_2_(*μ*_2_-SCH_2_C_6_H_4_-*p*-*^t^*Bu)_2_(*μ*_2_-SC_6_H_4_-*o*-P(O)(OPh)_2_]Cl (**17**) in CDCl_3_. |  | S17 | |
| **Figure S2c.** ^31^P NMR spectrum of [(*η*^6^-*p*-MeC_6_H_4_*^i^*Pr)_2_Ru_2_(*μ*_2_-SCH_2_C_6_H_4_-*p*-*^t^*Bu)_2_(*μ*_2_-SC_6_H_4_-*o*-P(O)(OPh)_2_]Cl (**17**) in CDCl_3_ |  | S17 | |
| **Figure S3a.** ^1^H NMR spectrum of [(*η*^6^-*p*-MeC_6_H_4_*^i^*Pr)_2_Ru_2_(*μ*_2_-SCH_2_C_6_H_4_-*p*-*^t^*Bu)_2_(*μ*_2_-SC_6_H_4_-*o*-BPin]Cl (**18**) in CDCl_3_. |  | S18 | |
| **Figure S3b.** ^13^C NMR spectrum of [(*η*^6^-*p*-MeC_6_H_4_*^i^*Pr)_2_Ru_2_(*μ*_2_-SCH_2_C_6_H_4_-*p*-*^t^*Bu)_2_(*μ*_2_-SC_6_H_4_-*o*-BPin]Cl (**18**) in CDCl_3_. |  | S18 | |
| **Figure S3c.** ^11^B NMR spectrum of [(*η*^6^-*p*-MeC_6_H_4_*^i^*Pr)_2_Ru_2_(*μ*_2_-SCH_2_C_6_H_4_-*p*-*^t^*Bu)_2_(*μ*_2_-SC_6_H_4_-*o*-BPin]Cl (**18**) in CDCl_3_. |  | S19 | |
| **Figure S4a.** ^1^H NMR spectrum of [(*η*^6^-*p*-MeC_6_H_4_*^i^*Pr)_2_Ru_2_(*μ*_2_-SCH_2_C_6_H_4_-*p*-*^t^*Bu)_2_(*μ*_2_-SC_6_H_4_-*o*-C(O)H]Cl (**19**) in CDCl_3_. |  | S19 | |
| **Figure S4b.** ^13^C NMR spectrum of [(*η*^6^-*p*-MeC_6_H_4_*^i^*Pr)_2_Ru_2_(*μ*_2_-SCH_2_C_6_H_4_-*p*-*^t^*Bu)_2_(*μ*_2_-SC_6_H_4_-*o*-C(O)H]Cl (**19**) in CDCl_3_. |  | S20 | |
| **Figure S5a.** ^1^H NMR spectrum of 6-mercapto-3,4-dihydroquinolin-2(1*H*)-one (**22a**) in DMDO-*d_6_* |  | S20 | |
| **Figure S5b.** ^13^C NMR spectrum of 6-mercapto-3,4-dihydroquinolin-2(1*H*)-one (**22a**) in DMDO-*d_6_* |  | S21 | |
| **Figure S6a.** ^1^H NMR spectrum of [(*η*^6^-*p*-MeC_6_H_4_*^i^*Pr)_2_Ru_2_(*μ*_2_-SCH_2_C_6_H_4_-*p*-*^t^*Bu)_2_(*μ*_2_-SR)]Cl (R = *(7)-* 1,3,4,5-tetrahydro-2H-benzo[*b*]azepin-2-one) (**20**) in CDCl_3_. |  | S21 | |
| **Figure S6b.** ^13^C NMR spectrum of [(*η*^6^-*p*-MeC_6_H_4_*^i^*Pr)_2_Ru_2_(*μ*_2_-SCH_2_C_6_H_4_-*p*-*^t^*Bu)_2_(*μ*_2_-SR)]Cl (R = *(7)-* 1,3,4,5-tetrahydro-2H-benzo[*b*]azepin-2-one) (**20**) in CDCl_3_. |  | S22 | |
| **Figure S7a.** ^1^H NMR spectrum of [(*η*^6^-*p*-MeC_6_H_4_Pr*^i^*)_2_Ru_2_(*μ*_2_-SR)_3_]Cl (R = *(7)* - 1,3,4,5-tetrahydro-2*H*-benzo[*b*]azepin-2-one) (**21**) in DMDO-*d_6_* |  | S22 | |
| **Figure S7b.** ^13^C NMR spectrum of [(*η*^6^-*p*-MeC_6_H_4_Pr*^i^*)_2_Ru_2_(*μ*_2_-SR)_3_]Cl (R = *(7)* - 1,3,4,5-tetrahydro-2*H*-benzo[*b*]azepin-2-one) (**21**) in DMDO-*d_6_* |  | S23 | |
| **Figure S8a.** ^1^H NMR spectrum of [(*η*^6^-*p*-MeC_6_H_4_*^i^*Pr)_2_Ru_2_(*μ*^2^-SCH_2_C_6_H_4_-*p*-*^t^*Bu)_2_(*μ*^2^-SR)]Cl (R = *(6)-* mercapto-3,4-dihydroquinolin-2(1*H*)-one) (**22**) in CDCl_3_ |  | S23 | |
| **Figure S8b.** ^13^C NMR spectrum of [(*η*^6^-*p*-MeC_6_H_4_*^i^*Pr)_2_Ru_2_(*μ*^2^-SCH_2_C_6_H_4_-*p*-*^t^*Bu)_2_(*μ*^2^-SR)]Cl (R = *(6)-* mercapto-3,4-dihydroquinolin-2(1*H*)-one) (**22**) in CDCl_3_. |  | S24 | |
| **Figure S9a.** ^1^H NMR spectrum of 7-mercapto-1,3,4,5-tetrahydro-2*H*-benzo[*b*]azepin-2-one (**23**) in DMDO-*d_6_.* |  | S24 | |
| **Figure S9b.** ^13^C NMR spectrum of 7-mercapto-1,3,4,5-tetrahydro-2*H*-benzo[*b*]azepin-2-one (**23**) in DMDO-*d_6_.* |  | S25 | |
| **Table S1.** Compilation of silver-, bismuth- and ruthenium-based metal complexes investigated for their antibacterial properties. |  | S26 | |
| **Figure S10.** MIC values of the 22 diruthenium complexes *included in this study* as a function of the*ir respective* molecular weight. The linear regression (trendline) for each bacterial species *is also* shown. |  | S28 | |
| **Figure S11.** Top: *C*ellular uptake of compounds **5**, **6**, **9**, **15** and **21** as a function of the MIC values against S. aureus 20. Bottom: *C*ellular uptake of compounds **5**, **6**, **9**, **15** and **21** as a function of the molecular weight. |  | S28 | |
| **Figure S12.** Z-cut photography under DAPI filter (445/450 nm) of *S. aureus* 20 cells treated with compound **15** for 3 h. |  | S29 | |
| **Figure S13.** Z-cut photography under GFP filter (525/550 nm) of *S. aureus* 20 cells treated with compound **15** for 3 h. |  | S30 | |
| **Figure S14.** Z-cut photography under Brightfield of *S. aureus* 20 cells treated with compound **15** for 3 h. |  | S31 | |
| **Figure S15.** Merged Z-cut photography with DAPI filter (445/450 nm), GFP filter (525/550 nm) and Brightfield of *S. aureus* 20 cells treated with compound **15** for 3 h.  **Table S2.** Effect of compounds **1-16** at 2.5 µM on HFF viability. |  | S32  S33    S34 | |
| **References** |  |  | |
|  | | |  |

**Experimental part - Chemistry**

1. **General**

Chemicals were purchased from Aldrich, Alfa Aesar, Acros Organics, ABCR, and TCI Chemicals and used without further purification. Reactions were performed under inert atmosphere (Ar) using Schlenk techniques with dry solvents (Acros Organics) preserved over molecular sieves. Commercial TMEDA used for the preparation of compounds **17a-19a** was purified by fractional distillation and filtration through activated ALOX(Neutral). ^1^H (400 MHz), ^13^C (101 MHz) and, where suitable, ^31^P NMR (121 MHz) and ^11^B NMR (96 MHz) spectra were recorded on a Bruker Avance II 400 spectrometer at 298 K. The chemical shifts are reported in parts per million (ppm) and referenced to residual solvent peaks (CDCl_3_, ^1^H *δ* 7.26 ppm, ^13^C{^1^H} *δ* 77.16 ppm. CD_2_Cl_2_ ^1^H *δ* 5.32 ppm, ^13^C{^1^H} *δ* 53.5 ppm, DMSO-*d*_6_ ^1^H 2.49 ppm, ^13^C{^1^H} *δ* 39.7 ppm), and coupling constants (*J*) are reported in hertz (Hz). High resolution electrospray ionization mass spectra (HR ESI-MS) were carried out by the Mass Spectrometry and Protein Analyses Services at DCBP and were obtained on a LTQ Orbitrap XL ESI (Thermo) operated in positive ion mode. Thermal elemental analyses were carried out by the Mass Spectrometry and Protein Analyses Services at DCBP and were obtained on a Flash 2000 Organic Elemental Analyzer (Thermo Scientific). Reactions were monitored by TLC using Merck TLC silica gel coated aluminium sheets 60 F254 and visualized with UV at 254 nm. Compounds were purified by column flash chromatography on silica gel using the elution systems indicated.

**Abbreviations**

ALOX: Aluminum oxide

DCE - 1,2-dichloroethane

THF - tetrahydrofuran

TMEDA - *N*,*N*,*N′*,*N′*-tetramethylethylenediamine

TBME - *tert*-butyl methyl ether

For the description of the NMR spectra: *Ar* – arene.

The synthesis, analysis and characterization of the diruthenium compounds **1-16** have been previously described.^1-6^

1. **Synthesis of compounds 17-19**
   1. **Synthesis of ligands 17a-19a**

The ligands used for the synthesis of diruthenium complexes **17-19** were prepared by initial ortho-lithiation of thiophenol (Scheme S1) and subsequent quenching of the corresponding dianion with diphenyl phosphorochloridate (for ligand **17a**), trimethyl borate (for ligand **18a**), and dimethylformamide (for ligand **19a**) by adapting previously reported protocols (53). Further transesterification of the dimethyl(2-mercaptophenyl)boronate (non-isolated) with pinacol provided ligand **18a** (2-(4,4,5,5-tetramethyl-1,3,2-dioxaborolan-2-yl)benzenethiol) (Scheme S1).

1.
2. **Scheme S1.** Synthesis of thiol ligands **17a-19a**.

***General procedure 1***

Adapted with modifications from a reported protocol^7^: To a dry sealed tube equipped with a stirring bar was added thiophenol (760 mg, 6.9 mmol) and TMEDA (2.3 mL, 15 mmol) under argon atmosphere. The mixture was stirred at 0 °C and then *n*-BuLi was added dropwise (6.7 mL, 2.5 M solution in hexane, 17.2 mmol). The reaction mixture was stirred at 0 °C for 2 h and further 14 h at r.t. The white precipitate formed was collected by centrifugation and washed with anhydrous hexane under argon atmosphere (3 X 5 mL). The precipitate was solubilized in anhydrous THF (6 mL) and cooled to -78 ºC. A solution of the corresponding electrophile (1.2 equiv in 1 mL of THF) was then added dropwise and the reaction mixture was stirred at -78 ºC for 3 h and further 12 h at r.t. The reaction was quenched by adding 10% aqueous HCl solution (10 mL) at 0 ºC, followed by 20 mL of water. The mixture obtained was extracted with chloroform (2 x 40 mL). The combined organic layers were then washed with brine (40 mL), dried over anhydrous Na_2_SO_4_, filtered, and concentrated under reduced pressure.

**Synthesis of diphenyl-(2-mercaptophenyl)phosphonate** (**17a)**

Prepared according to General procedure 1 using diphenyl phosphoryl chloride (1.7 mL, 8.3 mmol). The residue was purified by flash chromatography (Hept:EtOAc, 10:1 (v/v)) to provide **17a** as a viscous colorless oil (1.65 g, 70%).

**^1^H NMR (300 MHz, CD_2_Cl_2_) *δ*_H_, ppm:** 8.05-7.90 (1H, m, S-(*Ar*)C-C*H*-CH), 7.47-7.40 (2H, m), 7.38-7.30 (4H, m), 7.28-7.21 (6H, m), 7.21 – 7.16 (1H, m, S-(*Ar*)C-C-C*H*-CH), 5.30 (1H, d, (*Ar*)-S*H*, ^4^*J*_P,H_ = 1.1 Hz).

**^13^C NMR (75 MHz, CD_2_Cl_2_) *δ*_C_, ppm:** δ 150.71, 150.61, 139.01, 138.89, 135.76, 135.64, 133.81, 133.77, 131.39, 131.20, 130.17, 130.16, 125.74, 125.72, 125.42, 125.22, 120.97, 120.91.

**^31^P NMR (121 MHz, CD_2_Cl_2_) *δ*_P_, ppm:** 10.87.

**HR ESI-MS(+):** *m/z* found [M+H]^+^ 343.0550, calculated for C_18_H_16_O_3_PS 343.0552.

**Synthesis of 2-(4,4,5,5-tetramethyl-1,3,2-dioxaborolan-2-yl)benzenethiol (18a)**

Prepared according to General procedure 1 using trimethyl borate (0.95 mL, 8.3 mmol). The reaction crude was dissolved in 15 mL of anhydrous THF and then pinacol (1.7 mL, 13.8 mmol) and Na_2_SO_4_ anhydrous (2.9 g, 20.7 mmol) were added. The reaction mixture was stirred at r.t. for 12 h, and then the solids were filtered off. 40 mL of water were then added and the mixture was extracted with chloroform (2 x 40 mL). The combined organic layers were washed with brine (40 mL), dried over anhydrous Na_2_SO_4_, filtered, and concentrated under reduced pressure. The residue was purified by flash chromatography (Hept:EtOAc, 20:1 (v/v)) to provide **18a** as a viscous colorless oil (640 mg, 39%).

**^1^H NMR (400 MHz, CDCl_3_) *δ*_H_, ppm:**  7.78 – 7.71 (1H, m, S-(*Ar*)C-C-C*H*-CH), 7.30 – 7.18 (2H, m, S-(*Ar*)C-C*H*-C*H*-CH), 7.08 (1H, ddd, S-(*Ar*)C-C-CH-C*H*-CH, ^3^*J_H,H_* = 7.5 Hz, ^3^*J_H,H_* = 6.8, ^4^*J_H,H_* = 1.7 Hz), 5.20 (1H, (*Ar*)S*H*), 1.37 (12H, s, B(O-*C*-(C*H_3_*)_2_)_2_)

**^13^C NMR (101 MHz, CDCl_3_) *δ*_C_, ppm:** 140.66 (1C, S-*(Ar)C*-C), 137.32, (1C, S-*(Ar)*C-C-*C*H-CH), 131.45 (1C, S-(Ar)C-*C*H-CH), 128.61 (1C, S-(Ar)C-CH-*C*H-CH), 124.02 (1C, S-(Ar)C-CH-CH-*C*H-CH), 84.32 (2C, B(O-*C*-(CH_3_)_2_)_2_), 24.84 (4C, B(O-C-(*C*H_3_)_2_)_2_). **Note:** The signal corresponding to the *ipso* *sp^2^* carbon bonded to the boron atom does not appear.^8^

**^11^B NMR (128 MHz, CDCl_3_) *δ*_B_, ppm:** 30.92.

The spectroscopic data are in agreement with the published data.^9^

**Synthesis of 2-mercaptobenzaldehyde (19a)**

Prepared according to General procedure 1 with dimethylformamide (1.1 mL, 13.8 mmol). The residue was purified by flash chromatography (Pentane:TBME, 20:1 (v/v) to provide **19a** as an yellow oil (0.5 g, 52%).

**^1^H NMR (400 MHz, CDCl_3_) *δ*_H_, ppm:** 10.05 (1H, s, *H*(C=O)-*(Ar)*), 7.72 (1H, dd, S*-(Ar)*C-C-C*H*-CH), ^3^*J*_H,H_ = 7.5, ^4^*J*_H,H_  = 1.6 Hz), 7.38 (1H, ddd, S*-(Ar)*C-CH-C*H*-CH ^3^*J*_H,H_ = 8.6, ^3^*J*_H,H_ = 7.2, ^4^*J*_H,H_ = 1.6 Hz), 7.32 – 7.28 (2H, m, S*-(Ar)*C-C*H*-CH-C*H*-CH), 5.51 (1H, s, *H*S*-(Ar)*).

**^13^C NMR (101 MHz, CDCl_3_) *δ*_C_, ppm:** 192.89 (1C, *C*=O), 137.97 (1C, H(O=C)-*(Ar)C*-CH), 136.26 (1C, S-(*Ar*)C-*C*H-CH), 133.50 (1C, S*-(Ar)*C-CH-*C*H-CH), 131.36 (1C, S*-(Ar)C*-CH) 131.23 (1C, S*-(Ar)*C-C-CH-*C*H-CH), 125.06 (S-*(Ar)*C-C-*C*H-CH).

The spectroscopic data are in agreement with the published data.^10^

- 1. **Synthesis of the diruthenium complexes 17-19**

***General procedure 2***

The reactions were performed in sealed tubes. Dithiolato compound **4** (80 mg, 0.08 mmol) was dissolved in 10 mL of degassed DCE or CH_2_Cl_2_. The corresponding ligand **17a**-**19a** (5 equiv.) and triethylamine (62 *μ*L, 0.44 mmol) were then added. The mixture was stirred at 100 ºC (DCE, 10 mL) or 60 ºC (CH_2_Cl_2_, 10 mL) for 50 h. Then, the reaction crude was concentrated under reduced pressure and the residue was purified by flash chromatography.

**Synthesis of [(η^6^-p-MeC_6_H_4_^i^Pr)_2_Ru_2_(μ_2_-SCH_2_C_6_H_4_-p-^t^Bu)_2_(μ_2_-SC_6_H_4_-o-P(O)(OPh)_2_]Cl (17)**

Prepared according to General procedure 2 in DCE using diphenyl-(2-mercaptophenyl)phosphonate) **17a** (152 mg, 0.44 mmol). The residue was purified by flash chromatography (CH_2_Cl_2_:MeOH, 20:1 (v/v)) to afford **17** as an orange solid (63 mg, 58%).

**^1^H NMR (400 MHz, CDCl_3_)** ***δ*_H_, ppm:**  8.13 (1H, tt, *J_H,H_* = 7.1 Hz), 8.07 (2H, ddd, *J_H,H_* = 14.8, 7.8, 1.5 Hz), 7.74 (1H, t, *J* = 7.7 Hz), 7.54 (1H, ddd, *J* = 10.0, 6.7, 3.0 Hz), 7.47 – 7.40 (4H, m), 7.40 – 7.29 (10H, m), 7.22 (4H, dd, *J* = 8.0, 1.9 Hz), 5.36 (2H, dd, 2x(CH_3_)_2_CH-(*Ar*)C-C*H*-CH, ^3^*J*_H,H_ = 6.0 Hz, ^4^*J*_H,H_ = 1.2 Hz), 5.00 (2H, d, 2xCH_3_-(*Ar*)C-C*H*-CH, ^3^*J*_H,H_ = 6.4 Hz), 4.91 (2H, d, 2x(CH_3_)_2_CH-(*Ar*)C-C*H*-CH, ^3^*J*_H,H_ = 5.9 Hz), 4.53 (2H, d, 2xCH_3_-(*Ar*)C-C*H*, ^3^*J*_H,H_ = 5.9 Hz), 3.54, 3.46 (4H, s, 2xS-C*H_2_*(*Ar*)), 1.94 (2H, p, 2x(*Ar*)C*H*(CH_3_)_2_, ^3^*J*_H,H_ = 6.9 Hz), 1.64 (6H, s, 2xC*H_3_*(*Ar*)), 1.33 (18H, s, 2x(C*H_3_*)_3_C(*Ar*)), 0.91 (6H, d, *(Ar)*CH(C*H_3_*)_2_ ^3^*J*_H,H_ = 6.9 Hz), 0.87 (6H, d, *(Ar)*CH(C*H_3_*)_2_ ^3^*J*_H,H_ = 6.9 Hz).

**^13^C NMR (101 MHz, CDCl_3_)** ***δ*_C_, ppm:** 151.87, 151.80 (d, *J* = 15.0 Hz), 151.72, 151.06, 151.02 (d, *J* = 8.9 Hz), 150.97, 143.97, 143.92 (d, *J* = 9.8 Hz), 143.87, 136.96, 136.57, 135.94, 135.87 (d, *J* = 13.6 Hz), 135.80, 134.35, 133.41, 133.16, 133.12 (d, *J* = 9.3 Hz), 133.07, 131.41, 130.20, 129.46, 129.39 (d, *J* = 12.6 Hz), 129.33, 128.83, 128.75 (d, *J* = 14.6 Hz), 128.68, 125.63, 125.51, 120.43, 120.41 (d, *J* = 4.8 Hz), 120.38, 106.49 (2C, 2x(CH_3_)_2_CH-(*Ar*)*C*-CH), 101.29 (2C, 2xCH_3_-(*Ar*)*C*-CH), 84.78 (2C, 2x(CH_3_)_2_CH-(*Ar*)C-*C*H-CH), 83.82 (2C, 2xCH_3_*-(Ar)*C-*C*H-CH), 83.11 (2C, 2x(CH_3_)_2_CH-(*Ar*)C-*C*H-CH), 82.90 (2C, 2xCH_3_-(*Ar*)C-*C*H-CH), 40.38, 39.10 (2C, 2xS-*C*H_2_-*(Ar)*), 34.88 (2C, 2x(CH_3_)_3_-*C*-*(Ar)*), 31.56, 31.52 (6C, 2x(*C*H_3_)_3_C-*(Ar)*), 30.96 (2C, 2x(CH_3_)_2_*C*H-*(Ar)*), 23.31, 22.53 (4C, 2x(*C*H_3_)_2_CH-*(Ar)*), 18.29 (2C, 2x(*C*H_3_)-*(Ar)*).

**^31^P NMR (121 MHz, CD_2_Cl_2_)** ***δ*_P_, ppm:** 8.28.

**HR ESI-MS(+):** *m/z* found: 31171.2471 [M-Cl]^+^, calculated for C_60_H_72_O_3_PRu_2_S_3_ 1171.2473.

**Elemental analysis:** calcd (%) for C_60_H_72_O_3_PRu_2_S_3_Cl·2CH_3_OH: C 58.63, H 6.35; found: 58.59, H 7.04.

**Synthesis of [(η^6^-p-MeC_6_H_4_^i^Pr)_2_Ru_2_(μ_2_-SCH_2_C_6_H_4_-p-^t^Bu)_2_(μ_2_-SC_6_H_4_-o-BPin]Cl** **(18)**

Prepared according to General procedure 2 in DCE using 2-(4,4,5,5-tetramethyl-1,3,2-dioxaborolan-2-yl)benzenethiol **18a** (105 mg, 0.44 mmol). The residue was purified by flash chromatography (CH_2_Cl_2_:MeOH, 20:1 (v/v)) to afford **18** as an dark orange solid (40 mg, 41%).

**^1^H NMR (300 MHz, CDCl_3_) *δ*_H_, ppm:** 7.77 (1H, d, S-*(Ar)-*C-C-C*H*-CH, ^3^*J*_H-H_ = 7.1 Hz), 7.53 – 7.44 (2H, m, S-(Ar)C-C*H*-C*H*-CH), 7.45 – 7.36 (8H, m), 7.35 – 7.27 (1H, m, S-*(Ar)*C-C-CH-C*H*-CH), 5.23 – 5.12 (2H, m, 2x(CH_3_)_2_CH-(*Ar*)C-C*H*-CH), 5.08 (2H, d, 2xCH_3_-(*Ar*)C-C*H*-CH, ^3^*J*_H,H_ = 6.0 Hz), 4.84 – 4.76 (2H, m, 2x(CH_3_)_2_CH-(*Ar*)C-C*H*-CH), 4.62 – 4.54 (2H, d, 2xCH_3_-(*Ar*)C-C*H-*CH), 3.58, 3.45 (4H, s, 2xS-C*H_2_(Ar)*), 1.87 (2H, p, 2x(*Ar*)-C*H*(CH_3_)_2_, ^3^*J*_H,H_ = 6.8 Hz), 1.74 (6H, s, 2xC*H_3_*(*Ar*)), 1.49 (12H, 2x(*Ar*)-B((O-C-(C*H*_3_)_2_)_2_), 1.37 (9H, (C*H_3_*)_3_C*-(Ar)*), 1.34 (9H, s, (C*H_3_*)_3_C*-(Ar)*), 0.94 (6H, d, (C*H_3_*)_2_CH*-(Ar)*), ^3^*J*_H,H_ = 6.8 Hz), 0.89 (6H, d, (C*H_3_*)_2_CH*-(Ar)*), ^3^*J*_H,H_ = 6.9 Hz).

**^13^C NMR (101 MHz, CDCl_3_) *δ*_C_, ppm:** 152.07, 151.75 (2C, 2x(CH_3_)_3_C-(*Ar*)*C*-CH), 142.62 (1C, S-*(Ar)C*-C), 136.89, 136.81 (2C, 2xS-CH_2_-(*Ar*)*C*-CH), 133.19 (1C, S-*(Ar)*C-C-*C*H-CH), 132.69 (1C, S-(Ar)C-*C*H-CH), 131.00 (1C, S-(Ar)C-CH-*C*H-CH), 129.30, 129.26 (4C, 4xS-CH_2_-(*Ar*)C-*C*H-CH), 127.99 (1C, S-(Ar)C-CH-CH-*C*H-CH), 125.76, 125.53 (4C, 4x(CH_3_)_3_C(*Ar*)C-*C*H-CH), 107.48 (2C, 2x(CH_3_)_2_CH-(*Ar*)*C*-CH), 99.98 (2C, 2xCH_3_-(*Ar*)*C*-CH), 84.57 (2C, 2x(CH_3_)_2_CH-(*Ar*)C-*C*H-CH), 84.24 (2C, 2xCH_3_-(*Ar*)C-*C*H-CH), 83.95(2C, 2x(CH_3_)_2_CH-(*Ar*)C-*C*H-CH), 83.80(2C, 2xCH_3_-(*Ar*)C-*C*H-CH), 82.31 (2C, 2x(*Ar*)- B((O-*C*-(CH_3_)_2_)_2_), 39.87, 39.24 (2C, 2xS-*C*H_2_-(*Ar*)), 34.96, 34.90 (2C, 2x(CH_3_)_3_-*C-*(*Ar*)), 31.57 (6C, 2x(*C*H_3_)_3_C-(*Ar*)), 30.94, (2C, 2x(CH_3_)_2_*C*H-(*Ar*)) 25.37 (4C, 2x(*Ar*)-B(O-C-(*C*H_3_)_2_)_2_, 23.07, 22.91, (4C, 2x(*C*H_3_)_2_CH-(*Ar*)), 18.34 (2C, 2x(*C*H_3_)-(*Ar*)). **Note:** The signal corresponding to the *ipso* *sp^2^* carbon bonded to the boron atom does not appear.

**^11^B NMR (96 MHz, CDCl_3_) *δ*_B_, ppm:** 32.69.

**HR ESI-MS(+):** *m/z* found 1065.3036 [M-Cl]^+^, calculated for C_54_H_74_O_2_BRu_2_S_3_ 1065.3026.

**Elemental analysis:** calcd (%) for C_54_H_74_O_2_BRu_2_S_3_Cl·H_2_O: C 58.03, H 6.85; found: C 58.14, H 6.18.

**Synthesis of [(η^6^-p-MeC_6_H_4_^i^Pr)_2_Ru_2_(μ_2_-SCH_2_C_6_H_4_-p-^t^Bu)_2_(μ_2_-SC_6_H_4_-o-C(O)H]Cl (19)**

Prepared according to General procedure 2 in CH_2_Cl_2_ using 2-mercaptobenzaldehyde **19a** (61 mg, 0.44 mmol). The residue was purified by flash chromatography (CH_2_Cl_2_:MeOH, 20:1 (v/v)) to afford **19** as an orange solid (53 mg, 60%).

**^1^H NMR (400 MHz, CDCl_3_) *δ*_H_, ppm:**  11.11 (1H, s, *H*(C=O)-*(Ar)*), 8.00 (1H, dd, S*-(Ar)*C-C-C*H*-CH), ^3^*J*_H-H_ = 8.1, ^4^*J*_H-H_ = 1.1 Hz), 7.84 (1H, dd, S-(*Ar*)C-C*H*-CH, ^3^*J*_H-H_ = 7.7, ^4^*J*_H-H_ = 1.5 Hz), 7.71 (1H, td, S-(*Ar*)C-CH-CH-C*H*-CH, ^3^*J*_H-H_ = 7.6, ^4^*J*_H-H_ = 1.6 Hz), 7.51 – 7.36 (m, 9H), 5.13 (2H, dd, 2x(CH_3_)_2_CH-(*Ar*)C-C*H*-CH, ^3^*J*_H,H_ = 5.9, ^4^*J*_H,H_ = 1.2 Hz), 5.06 (2H, d, 2xCH_3_-(*Ar*)C-C*H*, ^3^*J*_H,H_ = 5.8 Hz), 5.01 (d, 2x(CH_3_)_2_CH-(*Ar*)C-C*H*-CH, ^3^*J*_H,H_ = 5.9 Hz), 4.66 – 4.60 (2H, m, 2x(CH_3_)_2_CH-(*Ar*)C-C*H*-CH), 3.67, 3.49 (4H, s, 2xS-C*H_2_(Ar)*), 1.89 (2H, hept, 2x(*Ar*)C*H*(CH_3_)_2_, ^3^*J*_H,H_ = 7.0 Hz), 1.76 (6H, s, 2xC*H_3_-(Ar)*), 1.36 (9H, s, (C*H_3_*)_3_C*-(Ar)*)1.31 (9H, s, (C*H_3_*)_3_C*-(Ar)*), 0.86 (12H, d, 2x(C*H_3_*)_2_CH*-(Ar)*, ^3^*J*_H,H_ = 6.9 Hz).

**^13^C NMR (101 MHz, CDCl_3_) *δ*_C_, ppm:**  192.00 (1C, *C*=O), 152.09, 151.77 (2C, 2x(CH_3_)_3_C-(*Ar*)*C*-CH), 142.42 (1C, H(O=C)-*(Ar)C*-CH), 138.69 (1C, S-(*Ar*)C-*C*H-CH), 136.81, 136.48 (2C, 2xS-CH_2_-(*Ar*)*C*-CH), 135.36 (1C, S*-(Ar)*C-CH-C*H*-CH), 135.34 H(O=C)-*(Ar)*C-CH-*C*H-CH) 129.52 (1C, S*-(Ar)C*-CH), 129.43, 129.34 (4C, 4xS-CH_2_-(*Ar*)C-*C*H-CH), 128.83 (1C, H(O=C)-*(Ar)*C-CH*-*C*H-*CH), 125.78, 125.55 (4C, 4x(CH_3_)_3_C(*Ar*)C-*C*H-CH), 107.26 (2C, 2x(CH_3_)_2_CH-(*Ar*)*C*-CH), 100.69 (2C, 2xCH_3_-(*Ar*)*C*-CH), 83.98 (2C, 2x(CH_3_)_2_CH-(*Ar*)C-*C*H-CH), 83.95 (2C, 2xCH_3_-(*Ar*)C-*C*H-CH), 83.64 (2C, 2x(CH_3_)_2_CH-(*Ar*)C-*C*H-CH), 82.65 (2C, 2xCH_3_-(*Ar*)C-*C*H-CH), 40.4, 39.37 (2C, 2xS-*C*H_2_-(*Ar*)), 34.93, 34.85 (2C, 2x(CH_3_)_3_-*C-*(*Ar*)), 31.52 (6C, 2x(*C*H_3_)_3_C-(*Ar*)), 31.02 (2C, 2x(CH_3_)_2_*C*H-(*Ar*)), 22.87, 22.82 (4C, 2x(*C*H_3_)_2_CH-(*Ar*)), 18.45 (2C, 2x(*C*H_3_)-(*Ar*)).

**HR ESI-MS(+):** *m/z* found 967.2127 [M-Cl]^+^, calculated for C_49_H_63_ORu_2_S_3_ 967.2123.

**Elemental analysis:** calcd (%) for C_49_H_63_ORu_2_S_3_Cl·0.5H_2_O: C 58.22, H 6.38; found C 58.0, H 6.34.

1. **Synthesis of compounds 20-22**
   1. **Synthesis of ligands and intermediates**
      1. **Synthesis of intermediates 22a’, 22a’’ and 23a’**

The ligands used for the synthesis of diruthenium complexes **20-22** were prepared by adapting literature protocols. **23** was obtained in two steps starting from 1-tetralone which was submitted to a nitrogen-insertion using NaN3 in acidic conditions (H2SO4), the 1,3,4,5-tetrahydro-2H-benzo[b]azepin-2-one (**23a’**) obtained as intermediate being subsequently submitted to a chlorosulfonation reaction and the in-situ PPh3-mediated reduction of the formed sulfonylchloride group in thiol. **22a** was synthetized following a three steps reaction sequence starting with the acylation of aniline with 3-chloropropanoyl chloride to obtain 3-chloro-N-phenylpropanamide (**22a”**), followed by an intramolecular Friedel-Crafts alkylation reaction in the presence of AlCl_3_ as Lewis acid allowing the obtainment of 3,4-dihydroquinolin-2(1H)-one (**22a’**), which was further submitted to a chlorosulfonation reaction and the in-situ reduction of the sulfonylchloride group in thiol using PPh3 (Scheme S2).

**Scheme S2.** Synthesis of thiol ligands **23** (top) and **22a** (bottom).

**Synthesis of 1,3,4,5-tetrahydro-2H-benzo[b]azepin-2-one (23a’)**

This compound was prepared by following a reported procedure.^11^ 1-Tetralone (5 g, 34.2 mmol) was dissolved in toluene (40 mL). Then, NaN_3_ (8.9 g, 137 mmol) was added and the mixture was cooled to 0 °C. Concentrated H_2_SO_4_ (95-98%, 16 ml) was slowly added and the reaction mixture was stirred at r.t. for 2 days. The formed solid was filtered off and washed with toluene (40 mL). The solid was suspended in water (40 mL) and the mixture was extracted with EtOAc (3 x 50mL). The combined organic layers were washed with brine (150mL), dried over anhydrous Na_2_SO_4_ and concentrated under reduced pressure. The desired product was obtained as pale yellow solid (2.8 g, 69%).

**^1^H NMR (300 MHz, CDCl_3_) *δ*_H_, ppm:** 8.72 (1H, s, N*H*(C=O)), 7.28-7.16 (2H, m, (C=O)NH-(*Ar*)C-C-C*H*-C*H*-CH), 7.14-7.07 (1H, m, (C=O)NH-(*Ar*)C-CH-C*H*-CH), 7.01 (1H, dd, (C=O)NH-(*Ar*)C-C*H*-CH), ^3^*J_H,H_* = 8.0, ^3^*J_H,H_ =* 1.4 Hz), 2.79 (2H, t, NH(C=O)-CH_2_-CH_2_-C*H*_2_-C(*Ar*)), ^3^*J_H,H_* = 7.1 Hz), 2.36 (2H, t, NH(C=O)-C*H*_2_-CH_2_, ^3^*J_H,H_* = 7.2 Hz, ), 2.31-2.15 (2H, m, NH(C=O)-CH_2_-C*H*_2_-CH_2_).

**^13^C NMR (75 MHz, CDCl_3_) *δ*_C_, ppm:** 175.79 (1C, NH(*C*=O)), 138.11 (1C, (C=O)NH-(*Ar*)*C*-CH), 134.33 (1C, (C=O)NH-(*Ar*)C-*C*-CH), 129.88 (1C, (C=O)NH-(*Ar*)C-C-*C*H-CH) 127.55 (1C, (C=O)NH-(*Ar*)C-C-CH-*C*H-CH), 125.66 (1C, (C=O)NH-(*Ar*)C-CH-*C*H-CH), 121.95 (C=O)NH-(*Ar*)C-*C*H-CH), 32.91 (1C, NH(C=O)-CH_2_-CH_2_-*C*H_2_-C(*Ar*)), 30.43 (1C, NH(C=O)-*C*H_2_-CH_2_), 28.67 (1C, NH(C=O)-*C*H_2_-*C*H_2_-CH_2_).

The spectroscopic data are in agreement with the published data.^11^

**Synthesis of 3-chloro-N-phenylpropanamide (22a’**’**)**

This compound was prepared by following a reported procedure.^12^ Aniline (9.3 g, 100 mmol) was dissolved in acetone (100 mL) and then potassium carbonate (20.8 g, 150 mmol) and water (200 mL) were added. The mixture was cooled to 0 ºC and 3-chloropropanoyl chloride (15.9 g, 125 mmol) was added dropwise. The resulting solution was stirred for 1 h at 0 °C, then a mixture of water/ice was added until precipitation occurred. The solid formed was filtered off and dried under reduced pressure. The desired product was obtained as a pale-yellow solid (13.8 g, 75%).

**^1^H NMR (400 MHz, CDCl_3_) *δ*_H_, ppm:** 7.85 (1H, s, N*H*(C=O)), 7.54-7.47 (2H, m, (C=O)NH-(*Ar*)C-C*H*-CH-CH-CH-C*H*), 7.30 (2H, t, (C=O)NH-(*Ar*)C-CH-C*H*-CH-C*H*-CH, ^3^*J*_H,H_ = 8.0 Hz), 7.17-7.06 (1H, m, (C=O)NH-(*Ar*)C-CH-CH-C*H*-CH), 3.85 (2H, t, NH(C=O)-CH_2_-C*H*_2_Cl, ^3^*J*_H,H_ = 6.4 Hz), 2.79 (2H, t, NH(C=O)-C*H*_2_-CH_2_Cl, ^3^*J*_H,H_ = 6.4 Hz),

**^13^C NMR (101 MHz, CDCl_3_) *δ*_C_, ppm:** 168.23 (1C, NH(*C*=O)), 137.56 (1C, NH(C=O)-(*Ar*)*C*-CH), 129.12 (2C, 2xNH(C=O)-(*Ar*)C-CH-*C*H-CH) 124.84 (1C, 2xNH(C=O)-(*Ar*)C-CH-CH-*C*H), 120.41 (2C, 2xNH(C=O)-(*Ar*)C-*C*H-CH), 40.45 (1C, NH(C=O)-CH_2_-*C*H_2_Cl), 40.04 (1C, NH(C=O)-*C*H_2_-CH_2_Cl).

The spectroscopic data are in agreement with the published data.^12^

**Synthesis of 3,4-dihydroquinolin-2(1*H*)-one** **(22a’)**

This compound was prepared by following a reported procedure.^13^ 3-chloro-*N*-phenylpropanamide (3 g, 16.3 mmol) and aluminum trichloride (8.7 g, 65.3 mmol) were mixed in a round bottom flask equipped with a stirring bar. The mixture was stirred at 150 ºC for 3 h (neat). After cooling to r.t., a mixture of water/ice was added until precipitation occurred. The formed solid was filtered off and dried under reduced pressure. The desired product was obtained as a pale-yellow solid (1.73 g, 72%).

**^1^H NMR (300 MHz, CDCl_3_) *δ*_H_, ppm:** 9.54 (1H, s, N*H*(C=O), 1H), 7.22 – 7.10 (2H, m, (C=O)NH-(*Ar*)C-C-C*H*-CH-C*H*-CH), 6.98 (1H, td, (C=O)NH-(*Ar*)C-C-CH-C*H*-CH ^3^*J*_H,H_ = 7.6 Hz, ^4^*J*_H,H_ = 1.2 Hz), 6.92 – 6.83 (1H, m, (C=O)NH-(*Ar*)C-C*H*-CH), 2.96 (2H, dd, NH(C=O)-CH_2_-C*H*_2_-C, ^3^*J*_H,H_ = 8.6, ^4^*J*_H,H_ = 6.5 Hz), 2.70 – 2.59 (2H, dd, NH(C=O)-C*H*_2_-CH_2_).

**^13^C NMR (75 MHz, CDCl_3_) *δ*_C_, ppm:** 172.59 (1C, NH(*C*=O), 137.45 (1C, (C=O)NH-(*Ar*)C-*C*-CH), 127.95, 127.61 (2C, (C=O)NH-(*Ar*)C-C-CH-*C*H-*C*H-CH), 123.67 (1C, (C=O)NH-(*Ar*)*C*-C), 123.13 (1C, (C=O)NH-(*Ar*)C-*C*H-CH), 115.75 (1C, (C=O)NH-(*Ar*)C-C-*C*H-CH), 30.79 (1C, NH(C=O)-*C*H_2_-CH_2_), 25.39 (1C, NH(C=O)-CH_2_-*C*H_2_-C).

The spectroscopic data are in agreement with the published data.^13^

- - 1. ***Synthesis of ligands 22a and 23***

***General procedure 3***

The following procedure was designed by modifying and adapting reported protocols.^14,15^ The lactams **22a’** and **23a** were placed in a two-neck round bottom flask equipped with a condenser. Chlorosulfonic acid (ClSO_3_H) (4 equiv.) was then added dropwise at 0 ºC, then the resulting reaction mixture was stirred for 1 h at 0 °C and further 1.5 h at 68 ºC. After cooling to r.t., the reaction mixture was poured dropwise on ice. The formed solid was filtered off, washed with water (50 mL), and dried under reduced pressure. The solid was then dissolved in THF (3 mL/mmol **23a** or **22a’**) and PPh_3_ (3.5 equiv.) and H_2_O (0.2 mL/ mmol **23a** or **22a’**) were added. The mixture was stirred for 90 min at 50ºC. After cooling to r.t., a solution of NaOH (2 M, 20 mL) was added to the reaction mixture and the phases were separated. The aqueous layer was washed with EtOAc (50 mL) and then a solution of HCl (2 M) was added until pH = 2 was reached. The solid formed was washed with H_2_O (50 mL) and dried under reduced pressure.

**Synthesis of 6-mercapto-3,4-dihydroquinolin-2(1*H*)-one (22a)**

Prepared according to the *General procedure 3* using 3,4-dihydroquinolin-2(1*H*)-one (1 g, 5.6 mmol), ClSO_3_H (1.5 mL, 22.3 mmol) and PPh_3_ (5.1 g, 19.5 mmol). **22a** was obtained as a pale-yellow solid (0.78 g, 64% over two steps).

**^1^H NMR (300 MHz, DMSO-*d*_6_) *δ*_H_, ppm:** 10.04 (1H, s, N*H*(C=O), 7.11 (1H, d, S-(*Ar*)C-C*H*-C, ^4^*J*_H,H_ = 2.2 Hz ), 7.07 (1H, dd, S-(*Ar*)C-C*H*-CH, ^3^*J*_H,H_ = 8.1, ^3^*J*_H,H_ = 2.2 Hz), 6.74 (1H, d, S-(*Ar*)C-CH-C*H*-C, ^3^*J*_H,H_ = 8.1 Hz), 5.11 (1H, (*Ar*)-S*H*), 2.81 (2H, t, NH(C=O)-CH_2_-C*H*_2_*-(Ar)*, ^3^*J*_H,H_ = 7.5 Hz), 2.41 (2H, t, NH(C=O)-C*H*_2_-CH_2_, ^3^*J*_H,H_ = 7.6 Hz).

**^13^C NMR (75 MHz, DMSO-*d*_6_) *δ*_C_, ppm:** 169.94 (1C, NH(*C*=O)), 136.11(1C, S-*(Ar*)C-CH-C-*C*-CH), 128.59 (1C, S-*(Ar*)C-*C*H-C), 127.93 (1C S-*(Ar*)C-*C*H-C), 124.65 (1C, S-(*Ar*)C-CH-*C*-C), 123.33 (1C, S-(*Ar*)*C*-CH), 115.74 (1C, S-(*Ar*)C-CH-*C*H-C), 30.24 (1C, NH(C=O)-*C*H_2-_CH_2_), 24.66 (1C, NH(C=O)-CH_2_-*C*H_2_*-(Ar)*).

**HR ESI-MS(+):** *m/z* found 180.0476 [M+H]^+^, calculated for C_9_H_10_ONS: 180.0478.

**Synthesis of 7-mercapto-1,3,4,5-tetrahydro-2*H*-benzo[*b*]azepin-2-one (23)**

Prepared according to the *General procedure 3* using 1,3,4,5-tetrahydro-2*H*-benzo[*b*]azepin-2-one (1 g, 6.2 mmol), ClSO_3_H (1.65 mL, 24.8 mmol) and PPh_3_ (5.7 g, 21.7 mmol). **23** was obtained as a white solid (0.9 g, 75% over two steps).

**^1^H NMR (300 MHz, DMSO-*d*_6_) *δ*_H_, ppm:** 9.43 (1H, s, N*H*(C=O)), 7.18 (1H, d, S-(*Ar*)C-C*H*-C, ^4^*J_H,H_* = 2.2 Hz), 7.14 (1H, dd, S-(*Ar*)C-C*H*-CH, ^3^*J*_H,H_ = 8.1 Hz, ^4^*J*_H,H_ = 2.2 Hz), 6.84 (1H, d, S-(*Ar*)C-CH-C*H*-C, ^3^*J_H,H_* = 8.1 Hz), 5.28 (1H, s, (*Ar*)-S*H*), 2.61 (2H, t, NH(C=O)-CH_2_-CH_2_-C*H*_2_-(*Ar*), ^3^*J_H,H_* = 6.7 Hz), 2.18 – 1.96 (4H, m, NH(C=O)-C*H*_2_-C*H*_2_-CH_2_).

**^13^C NMR (75 MHz, DMSO-*d*_6_) *δ*_C_, ppm:**  173.10 (1C, NH(*C*=O)), 136.31 (1C, S-*(Ar*)C-CH-C-*C*-CH), 134.60 (1C, S-(*Ar*)C-CH-*C*-C), 129.76 (1C, S-(*Ar*)C-*C*H-C), 127.48 (1C, S-(*Ar*)C-*C*H-CH), 127.18 (1C, S-(*Ar*)*C*-CH), 122.31 (1C, S-(*Ar*)C-CH-*C*H-C), 32.76 (1C, NH(C=O)-*C*H_2_-CH_2_), 29.70 (1C, NH(C=O)-CH_2_-CH_2_-*C*H_2_-*(Ar)*), 27.69 (1C, NH(C=O)-CH_2_-*C*H_2_-CH_2_).

**HR ESI-MS(+):** *m/z* found 194.0632 [M+H]^+^, calculated for C_10_H_12_ONS 194.0634.

- 1. **Synthesis of diruthenium complexes 20, 21 and 22**

**Synthesis of [(η^6^-p-MeC_6_H_4_^i^Pr)_2_Ru_2_(μ_2_-SCH_2_C_6_H_4_-p-^t^Bu)_2_(μ_2_-SR)]Cl (RSH = 7-mercapto-1,3,4,5-tetrahydro-2H-benzo[b]azepin-2-one)** **(20)**

The reaction was performed in a sealed tube. Dithiolato compound **4** (80 mg, 0.08 mmol) and 7-mercapto-1,3,4,5-tetrahydro-2H-benzo[b]azepin-2-one **23** (51 mg, 0.26 mmol) were dissolved in degassed DCE (15 mL). The resulting solution was stirred at 80 ºC for 12 h, and then the reaction mixture was concentrated under reduced pressure. The residue was then purified by flash chromatography (CH_2_Cl_2_:MeOH, 15:1 (v/v)) to afford **20** as an orange solid (65 mg, 67%).

**^1^H NMR (300 MHz, CDCl_3_)** ***δ*_H_, ppm:** 8.29 (1H, s, *H*(C=O)-*(Ar)*), 7.66 (2H d ^3^*J*_H,H_ = 7.3 Hz); 7.50-7.37(8H, m, 2xS-CH_2_-(*Ar)*C-(C*H*-C*H*)_2_), 5.18-5.10 (2H, m, 2xS-(Ar)C-C*H*-C), 5.02 (2H, 2x(CH_3_)_2_CH-(*Ar*)C-C*H*-CH, ^3^*J*_H,H_ = 5.9 Hz), 4.97(2H, d, 2xCH_3_-(*Ar*)C-C*H*-CH, ^3^*J*_H,H_ = 5.8 Hz), 4.59 (2H, 2x(CH_3_)_2_CH-(*Ar*)C-C*H*-CH, ^3^*J*_H,H_ = 5.9 Hz), 3.60, 3.42 (4H, s, 2xS-C*H_2_(Ar)*), 2.89 (2H, d, NH(C=O)CH_2_-CH_2_-C*H*_2_-C, ^3^*J*_H,H_ = 7.0 Hz), 2.30 (2H, d, NH(C=O)C*H*_2_-CH_2_ ^3^*J*_H,H_ = 7.1 Hz); 1.95-1.84 (2H, 2x(*Ar*)C*H*(CH_3_)_2_), 1.78 (6H, s, 2xC*H_3_-(Ar)*), 1.36, 1.33 (18H, s, 2x(C*H_3_*)_3_C*-(Ar)*), 0.93 (6H, d, 2x(C*H_3_*)_2_CH*-(Ar)*, ^3^*J*_H,H_ = 6.9 Hz), 0.89 (6H, d, 2x(C*H_3_*)_2_CH*-(Ar)*, ^3^*J*_H,H_ = 6.9 Hz).

**^13^C NMR (101 MHz, CDCl_3_) *δ*_C_, ppm:** 174.63 (1C, NH*C*=O), 151.88, 151.79 (2C, 2x(CH_3_)_3_C-(*Ar*)*C*-CH), 138.97 (1C, (C=O)NH-(*Ar*)*C*-CH), 136.73, 136.70 (2C, 2xS-CH_2_-(*Ar*)*C*-CH-CH-C-C(CH_3_)_3_), 134.71 (1C, S-(Ar)*C*-CH-CH), 133.97 (1C, S-(*Ar*)C-*C*H-CH), 133.74 (1C, S-(*Ar*)C-CH-*C*-C), 131.89 (1C, S-(*Ar*)C-*C*H-C), 129.36 , 129.17 (4C, 4xS-CH_2_-(*Ar*)C-*C*H-CH), 125.67, 125.51 (4C, 4x(CH_3_)_3_-(*Ar*)C-*C*H-CH), 122.95 (1C, S-(*Ar*)C-CH-*C*H-C), 106.86 (2C, 2x(CH_3_)_2_CH-(*Ar*)*C*-CH), 100.75 (2C, 2xCH_3_-(*Ar*)*C*-CH), 84.33 (2C, 2x(CH_3_)_2_CH-(*Ar*)C-*C*H-CH), 83.67 (2C, 2xCH_3_-(*Ar*)C-*C*H-CH), 83.58 (2C, 2x(CH_3_)_2_CH-(*Ar*)C-*C*H-CH), 82.57 (2C, 2xCH_3_-(*Ar*)C-*C*H-CH), 40.04, 39.46 (2C, 2xS-*C*H_2_-(*Ar*)), 34.87, 34.84 (2C, 2x(CH_3_)_3_*C*-(*Ar*)), 33.17 (1C, NH(C=O)-CH_2_-CH_2_-*C*H_2_-C(*Ar*)), 31.52, 31.50 (6C, 2x(*C*H_3_)_3_C-(*Ar*)), 30.91 (1C, 2x(CH_3_)_2_*C*H-(*Ar*)), 30.51(1C, NH(C=O)-*C*H_2_-CH_2_) 28.58 (1C, NH(C=O)-CH_2_-*C*H_2_-*C*H_2_), 23.16, 22.78, (4C, 2x(*C*H_3_)_2_CH-(*Ar*)) 18.32 (2C, 2x(*C*H_3_)-(*Ar*)).

**HR ESI-MS(+):** *m/z* found 1022.2545 [M-Cl]^+^, calculated for C_52_H_68_ONRu_2_S_3_ 1022.2545.

**Elemental analysis:** calcd (%) for C_52_H_68_ONRu_2_S_3_Cl·CH_2_Cl_2_: C 55.75, H 6.18, N 1.23; found: C 55.95, H, 6.32, N 1.16.

# **Synthesis of [(*η*^6^-*p*-MeC_6_H_4_*^i^*Pr)_2_Ru_2_(*μ*_2_-SR)_3_]Cl (RSH = 7-mercapto-1,3,4,5-tetrahydro-2*H*-benzo[*b*]azepin-2-one) (21)**

Commercially available [Ru(*p*-cymene)Cl_2_]_2_ (dichloro(*p*-cymene)ruthenium(II) dimer) (50 mg, 0.08 mmol), 7-mercapto-1,3,4,5-tetrahydro-2*H*-benzo[*b*]azepin-2-one **23** (78 mg, 0.4 mmol) and K_2_CO_3_ (45 mg, 0,32 mmol) were mixed in degassed ethanol. The resulting mixture was refluxed for 12 h, and then the reaction crude was concentrated under reduced pressure and the residue was purified by flash chromatography (CH_2_Cl_2_:MeOH, 15:1(v/v)) to afford **21** as an orange solid (53 mg, 55%).

**^1^H NMR (400 MHz, DMSO-*d*_6_) *δ*_H_, ppm:**  9.68 (3H, s, 3xN*H-*(C=O)), 7.82 (3H, d, 3xS-(*Ar*)C-C*H*-C, ^4^*J*_H,H_ = 2.3 Hz,), 7.69 (3H, dd, 3xS-(*Ar*)C-C*H*-CH, ^3^*J*_H,H_ = 8.2, ^4^*J*_H,H_ = 2.3 Hz), 6.98 (3H, d, 3xS-(*Ar*)C-CH-C*H*-C, ^3^*J*_H,H_ = 8.2 Hz), 5.71 (2H, d, 2x(CH_3_)_2_CH-(*Ar*)C-C*H*-CH, ^3^*J*_H,H_ = 5.8 Hz), 5.48 (2H, d, 2xCH_3_-(*Ar*)C-C*H*-CH, ^3^*J*_H,H_ = 6.0 Hz), 5.38 (2H, d, 2x(CH_3_)_2_CH-(*Ar*)C-C*H*-CH, ^3^*J*_H,H_ = 6.0 Hz), 5.33 (2H, d, 2xCH_3_-(*Ar*)C-C*H*-CH, ^3^*J*_H,H_ = 5.8 Hz), 2.81 (6H, m, 3xNH(C=O)CH_2_-CH_2_-C*H_2_*-C), 2.19 (12H, brs, 3xNH(C=O)C*H_2_*-C*H_2_*-CH_2_), 1.88 (2H, hept, 2x (CH_3_)_2_C*H*(*Ar*) ^3^*J*_H,H_ = 6.7 Hz), 1.59 (6H, s, 2xC*H_3_*(*Ar*)), 0.85 (6H, d, (C*H_3_*)_2_CH*(Ar)*, ^3^*J*_H,H_ = 6.9 Hz), 0.72 (6H, d, (C*H_3_*)_2_CH*(Ar),* ^3^*J*_H,H_ = 6.8 Hz).

**^13^C NMR (101 MHz, DMSO-*d*_6_)** ***δ*_C_, ppm:**  173.05 (3C, 3xNH-(*C*=O)), 138.74 (3C, 3xS-(*Ar*)C-CH-CH-*C*), 133.96 (3C, 3xS-(*Ar*)C-CH-*C*), 133.54 (3C, 3xS-(*Ar*)C-*C*H-CH-C), 133.08 (3C, 3xS-(*Ar*)*C*-CH), 131.14 (3C, 3xS-(*Ar*)C-*C*H-C), 121.82 (3C, 3xS-(*Ar*)C-CH-*C*H-C), 105.76 (2C, 2x(CH_3_)_2_CH-(*Ar*)*C*-CH), 99.98 (2C, 2xCH_3_-(*Ar*)*C*-CH), 85.79 (2C, 2x(CH_3_)_2_CH-(*Ar*)C-*C*H-CH), 84.83 (2C, 2xCH_3_-(*Ar*)C-*C*H-CH), 84.23 (2C, 2x(CH_3_)_2_CH-(*Ar*)C-*C*H-CH), 83.86 (2C, 2xCH_3_-(*Ar*)C-*C*H-CH), 32.95 (3C, 3xNH-(C=O)-CH_2_-CH_2_-*C*H_2_-C), 30.13 (3C, 3xNH-(C=O)-*C*H_2_-CH_2_-CH_2_-C), 29.86 (2C, 2x(CH_3_)_2_*C*H-(*Ar*)), 27.90 (3C, 3xNH-(C=O)-CH_2_-*C*H_2_-CH_2_-C), 22.01, 21.48 (2C, 2x(*C*H_3_)_2_CH-(*Ar*)), 17.15 (2C, 2x(*C*H_3_)-(*Ar*)).

**HR ESI-MS(+):** *m/z* found 1048.1725 [M-Cl]^+^, calculated for C_50_H_58_O_3_N_3_Ru_2_S_3_ 1048.1722.

**Elemental analysis:** calcd (%) for C_50_H_58_O_3_N_3_Ru_2_S_3_Cl·2H_2_O: C 53.68, H 5.59, N 3.76; found: C 53.49, H 5.67, N 3.2.

**Synthesis of [(η^6^-p-MeC_6_H_4_^i^Pr)_2_Ru_2_(μ_2_-SCH_2_C_6_H_4_-p-^t^Bu)_2_(μ_2_-SR)]Cl (RSH = 6-mercapto-3,4-dihydroquinolin-2(1H)-one) (22)**

Reaction performed in a sealed tube. Dithiolato compound **4** (80 mg, 0.08 mmol) and 6-mercapto-3,4-dihydroquinolin-2(1H)-one **22a** (47 mg, 0.26 mmol) were dissolved in degassed DCE (15 mL). The resulting solution was stirred at 80ºC for 12 h, then the reaction mixture was concentrated under reduced pressure and the residue purified was by flash chromatography (CH_2_Cl_2_:MeOH, 15:1 (v/v)) to afford **22** as an orange solid (52 mg, 56%).

**^1^H NMR (300 MHz, CDCl_3_) *δ*_H_, ppm:** 9.37 (1H, s, *H*(C=O)-*(Ar)*)), 7.57 (2H, dd, ^3^*J*_H,H_ = 7.6, ^4^*J*_H,H_ = 1.2 Hz), 7.53 – 7.37 (9H, m), 7.29 (1H, brs), 5.10 (2H, d, 2x(CH_3_)_2_CH-(*Ar*)C-C*H*-CH, ^3^*J*_H,H_ = 5.8 Hz), 4.98 (d, 2xCH_3_-(*Ar*)C-C*H*-CH, ^3^*J*_H,H_ = 5.9 Hz), 4.90 (d, 2x(CH_3_)_2_CH-(*Ar*)C-C*H*-CH, ^3^*J*_H,H_ = 5.8 Hz), 4.64 – 4.56 (2H, m, 2x(CH_3_)_2_CH-(*Ar*)C-C*H*-CH), 3.58, 3.40 (4H, s, 2xS-C*H_2_(Ar)*), 3.05 (2H, NH(C=O)CH_2_-C*H_2_*-C, ^3^*J*_H,H_ = 7.6 Hz), 2.65 (2H, dd, NH(C=O)C*H_2_*-CH_2_-C, ^3^*J*_H,H_ = 8.5, 6.5 Hz), 1.95 (2H, hept, 2x(*Ar*)C*H*(CH_3_)_2_, ^3^*J*_H,H_ = 6.9 Hz), 1.75 (6H, s, 2xC*H_3_-(Ar)*), 1.37 (9H, s, (C*H_3_*)_3_C*-(Ar)*),1.34 (9H, s, (C*H_3_*)_3_C*-(Ar)*), (12H, d, 2x(C*H_3_*)_2_CH*-(Ar)*, ^3^*J*_H,H_ = 6.8 Hz,), 0.92 (12H, d, 2x(C*H_3_*)_2_CH*-(Ar)*, ^3^*J*_H,H_ = 6.9 Hz).

**^13^C NMR (101 MHz, CDCl_3_) *δ*_C_, ppm****:**  170.87 (1C, NH*C*=O), 151.93, 151.84 (2C, 2x(CH_3_)_3_C-(*Ar*)*C*-CH), 138.83 (1C, S-(*Ar*)*C*-CH), 136.85, 136.79 (2C, 2xS-CH_2_-(*Ar*)*C*-CH), 132.01 (1C, S-(*Ar*)C-CH-*C*-CH) 131.83 (1C, (C=O)N-*(Ar)*C-CH, 130.45 (1C, S-(*Ar*)C-CH-*C*H-C-N(C=O), 129.40, 129.22 (4C, 4xS-CH_2_-(*Ar*)C-*C*H-CH), 125.72, 125.58 (4C, 4x(CH_3_)_3_C(*Ar*)C-*C*H-CH), 124.25 (1C, S-(*Ar*)C-C*H*-CH), 117.14 (1C, S-(*Ar*)C-*C*H-C), 107.15 (2C, 2x(CH_3_)_2_CH-(*Ar*)*C*-CH), 100.55 (2C, 2xCH_3_-(*Ar*)*C*-CH), 84.08 (2C, 2x(CH_3_)_2_CH-(*Ar*)C-*C*H-CH), 83.87 (2C, 2xCH_3_-(*Ar*)C-*C*H-CH), 83.79 (2C, 2x(CH_3_)_2_CH-(*Ar*)C-*C*H-CH), 82.50 (2C, 2xCH_3_-(*Ar*)C-*C*H-CH), 40.05, 39.39 (2C, 2xS-*C*H_2_-(*Ar*)), 34.94, 34.91 (2C, 2x(CH_3_)_3_*C*-(*Ar*)), 31.56 (6C, 2x(*C*H_3_)_3_C-(*Ar*)), 31.03 (2C, 2x(CH_3_)_2_*C*H-(*Ar*)), 30.96 (1C, NH(C=O)*C*H_2_-CH_2_-C) 25.62 (1C, NH(C=O)CH_2_-*C*H_2_-C), 23.21, 22.84 (4C, 2x(*C*H_3_)_2_CH-(*Ar*)), 18.34 (2C, 2x(*C*H_3_)-(*Ar*)).

**HR ESI-MS(+):** *m/z* found 1008.2402 [M-Cl]^+^, calculated for C_51_H_66_ONRu_2_S_3_ 1008.2388.

**Elemental analysis:** calcd (%) for C_51_H_66_ONRu_2_S_3_Cl·H_2_O: C 57.74, H 6.46, N 1.32; found: C 57.34, H 6.61, N 1.18

1. **NMR spectra of compounds 17a, 17, 18, 19, 22a, 20, 21, 22 and 23**

**Diphenyl-(2-mercaptophenyl)phosphonate** **(17a)**

**Figure S1a.** ^1^H NMR spectrum of diphenyl-(2-mercaptophenyl)phosphonate (**17a**) in CD_2_Cl_2_.

**Figure S1b.** ^13^C NMR spectrum of diphenyl-(2-mercaptophenyl)phosphonate (**17a**) in CD_2_Cl_2_.

**Figure S1c.** ^31^P NMR spectrum of diphenyl-(2-mercaptophenyl)phosphonate (**17a**) in CD_2_Cl_2_.

**[(*η*^6^-*p*-MeC_6_H_4_*^i^*Pr)_2_Ru_2_(*μ*_2_-SCH_2_C_6_H_4_-*p*-*^t^*Bu)_2_(*μ*_2_-SC_6_H_4_-*o*-P(O)(OPh)_2_]Cl (17)**

**Figure S2a.** ^1^H NMR spectrum of [(*η*^6^-*p*-MeC_6_H_4_*^i^*Pr)_2_Ru_2_(*μ*_2_-SCH_2_C_6_H_4_-*p*-*^t^*Bu)_2_(*μ*_2_-SC_6_H_4_-*o*-P(O)(OPh)_2_]Cl (**17**) in CDCl_3_.

**Figure S2b.** ^13^C NMR spectrum of [(*η*^6^-*p*-MeC_6_H_4_*^i^*Pr)_2_Ru_2_(*μ*_2_-SCH_2_C_6_H_4_-*p*-*^t^*Bu)_2_(*μ*_2_-SC_6_H_4_-*o*-P(O)(OPh)_2_]Cl (**17**) in CDCl_3_.

**Figure S2c.** ^31^P NMR spectrum of [(*η*^6^-*p*-MeC_6_H_4_*^i^*Pr)_2_Ru_2_(*μ*_2_-SCH_2_C_6_H_4_-*p*-*^t^*Bu)_2_(*μ*_2_-SC_6_H_4_-*o*-P(O)(OPh)_2_]Cl (**17**) in CDCl_3_.

**[(*η*^6^-*p*-MeC_6_H_4_*^i^*Pr)_2_Ru_2_(*μ*_2_-SCH_2_C_6_H_4_-*p*-*^t^*Bu)_2_(*μ*_2_-SC_6_H_4_-*o*-BPin]Cl** **(18)**

**Figure S3a.** ^1^H NMR spectrum of [(*η*^6^-*p*-MeC_6_H_4_*^i^*Pr)_2_Ru_2_(*μ*_2_-SCH_2_C_6_H_4_-*p*-*^t^*Bu)_2_(*μ*_2_-SC_6_H_4_-*o*-BPin]Cl (**18**) in CDCl_3_.

**Figure S3b.** ^13^C NMR spectrum of [(*η*^6^-*p*-MeC_6_H_4_*^i^*Pr)_2_Ru_2_(*μ*_2_-SCH_2_C_6_H_4_-*p*-*^t^*Bu)_2_(*μ*_2_-SC_6_H_4_-*o*-BPin]Cl (**18**) in CDCl_3_.

**Figure S3c.** ^11^B NMR spectrum of [(*η*^6^-*p*-MeC_6_H_4_*^i^*Pr)_2_Ru_2_(*μ*_2_-SCH_2_C_6_H_4_-*p*-*^t^*Bu)_2_(*μ*_2_-SC_6_H_4_-*o*-BPin]Cl (**18**) in CDCl_3_.

**[(*η*^6^-*p*-MeC_6_H_4_*^i^*Pr)_2_Ru_2_(*μ*_2_-SCH_2_C_6_H_4_-*p*-*^t^*Bu)_2_(*μ*_2_-SC_6_H_4_-*o*-C(O)H]Cl (19)**

**Figure S4a.** ^1^H NMR spectrum of [(*η*^6^-*p*-MeC_6_H_4_*^i^*Pr)_2_Ru_2_(*μ*_2_-SCH_2_C_6_H_4_-*p*-*^t^*Bu)_2_(*μ*_2_-SC_6_H_4_-*o*-C(O)H]Cl (**19**) in CDCl_3_.

**Figure S4b.** ^13^C NMR spectrum of [(*η*^6^-*p*-MeC_6_H_4_*^i^*Pr)_2_Ru_2_(*μ*_2_-SCH_2_C_6_H_4_-*p*-*^t^*Bu)_2_(*μ*_2_-SC_6_H_4_-*o*-C(O)H]Cl (**19**) in CDCl_3_.

**6-Mercapto-3,4-dihydroquinolin-2(1*H*)-one (22a)**

**Figure S5a.** ^1^H NMR spectrum of 6-mercapto-3,4-dihydroquinolin-2(1*H*)-one (**22a**) in DMDO-*d_6_.*

**Figure S5b.** ^13^C NMR spectrum of 6-mercapto-3,4-dihydroquinolin-2(1*H*)-one (**22a**) in DMDO-*d_6_.*

**[(*η*^6^-*p*-MeC_6_H_4_*^i^*Pr)_2_Ru_2_(*μ*_2_-SCH_2_C_6_H_4_-*p*-*^t^*Bu)_2_(*μ*_2_-SR)]Cl (R-SH = 7-mercapto1,3,4,5-tetrahydro-2*H*-benzo[*b*]azepin-2-one) (20)**


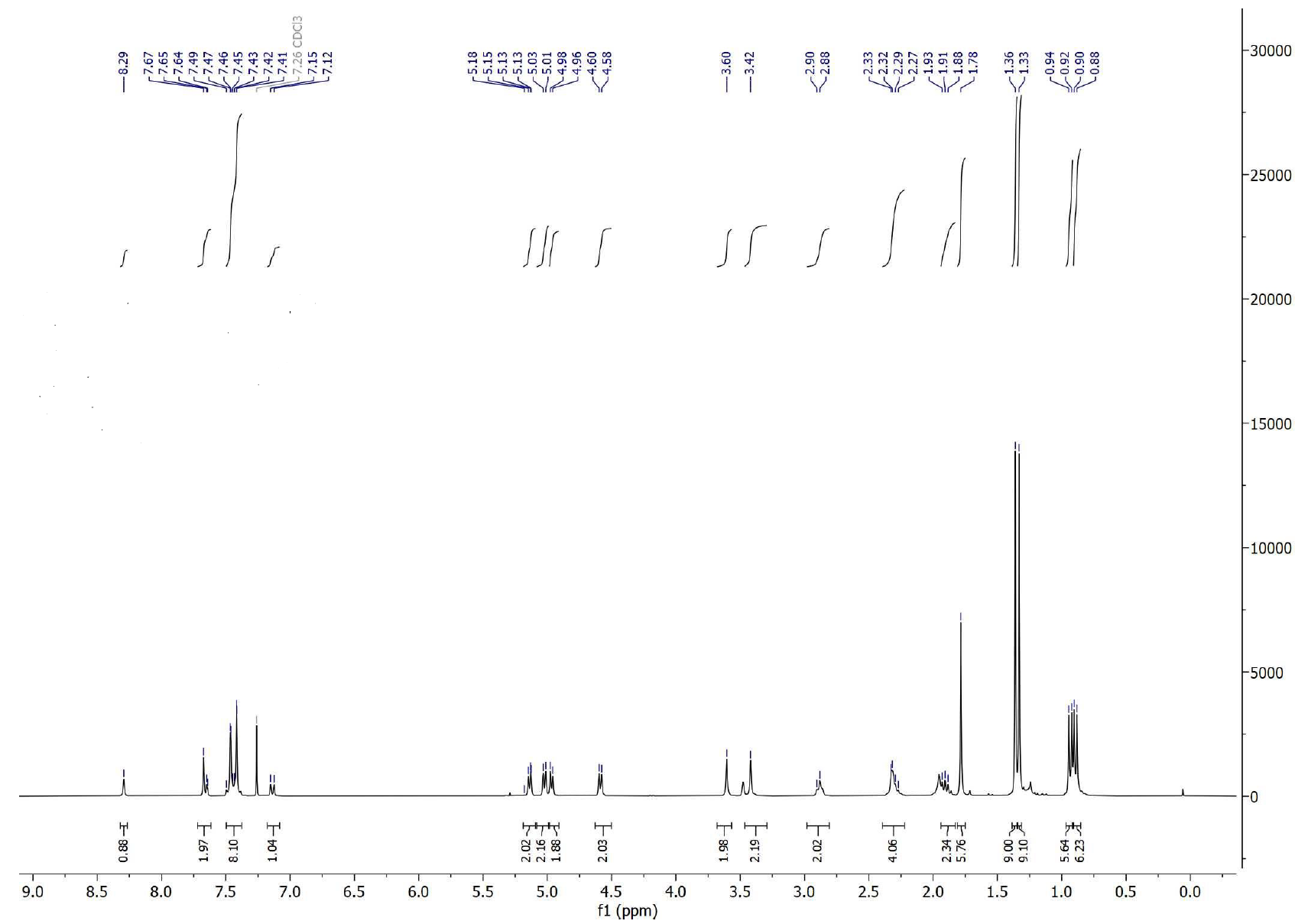


**Figure S6a.** ^1^H NMR spectrum of [(*η*^6^-*p*-MeC_6_H_4_*^i^*Pr)_2_Ru_2_(*μ*_2_-SCH_2_C_6_H_4_-*p*-*^t^*Bu)_2_(*μ*_2_-SR)]Cl ((R-SH = 7-mercapto1,3,4,5-tetrahydro-2*H*-benzo[*b*]azepin-2-one) (**20**) in CDCl_3_.

**Figure S6b.** ^13^C NMR spectrum of [(*η*^6^-*p*-MeC_6_H_4_*^i^*Pr)_2_Ru_2_(*μ*_2_-SCH_2_C_6_H_4_-*p*-*^t^*Bu)_2_(*μ*_2_-SR)]Cl (R-SH = 7-mercapto1,3,4,5-tetrahydro-2*H*-benzo[*b*]azepin-2-one) (**20**) in CDCl_3_.

**[(*η*^6^-*p*-MeC_6_H_4_Pr*^i^*)_2_Ru_2_(*μ*_2_-SR)_3_]Cl (R-SH = 7-mercapto1,3,4,5-tetrahydro-2*H*-benzo[*b*]azepin-2-one) (21)**

**Figure S7a.** ^1^H NMR spectrum of [(*η*^6^-*p*-MeC_6_H_4_Pr*^i^*)_2_Ru_2_(*μ*_2_-SR)_3_]Cl (R-SH = 7-mercapto1,3,4,5-tetrahydro-2*H*-benzo[*b*]azepin-2-one) (**21** )in DMDO-*d_6_*

**Figure S7b.** ^13^C NMR spectrum of [(*η*^6^-*p*-MeC_6_H_4_Pr*^i^*)_2_Ru_2_(*μ*_2_-SR)_3_]Cl (R-SH = 7-mercapto1,3,4,5-tetrahydro-2*H*-benzo[*b*]azepin-2-one) (**21**) in DMDO-*d_6_*.

**[(*η*^6^-*p*-MeC_6_H_4_*^i^*Pr)_2_Ru_2_(*μ*^2^-SCH_2_C_6_H_4_-*p*-*^t^*Bu)_2_(*μ*^2^-SR)]Cl (R-SH = 6-mercapto-3,4-dihydroquinolin**

**-2(1*H*)-one) (22)**

**Figure S8a.** ^1^H NMR spectrum of [(*η*^6^-*p*-MeC_6_H_4_*^i^*Pr)_2_Ru_2_(*μ*^2^-SCH_2_C_6_H_4_-*p*-*^t^*Bu)_2_(*μ*^2^-SR)]Cl (R-SH = 6-mercapto-3,4-dihydroquinolin-2(1*H*)-one) (**22**) in CDCl_3_.

**Figure S8b.** ^13^C NMR spectrum of [(*η*^6^-*p*-MeC_6_H_4_*^i^*Pr)_2_Ru_2_(*μ*^2^-SCH_2_C_6_H_4_-*p*-*^t^*Bu)_2_(*μ*^2^-SR)]Cl (R-SH = 6-mercapto-3,4-dihydroquinolin-2(1*H*)-one) (**22**) in CDCl_3_.

**7-Mercapto-1,3,4,5-tetrahydro-2*H*-benzo[*b*]azepin-2-one (23)**

**Figure S9a.** ^1^H NMR spectrum of 7-mercapto-1,3,4,5-tetrahydro-2*H*-benzo[*b*]azepin-2-one (**23**) in DMDO-*d_6_.*

**Figure S9b.** ^13^C NMR spectrum of 7-mercapto-1,3,4,5-tetrahydro-2*H*-benzo[*b*]azepin-2-one (**23**) in DMDO-*d_6._*

**Table S1.** Compilation of **s**ilver-, bismuth- and ruthenium-based metal complexes investigated for their antibacterial properties. The references are provided in the main manuscript.

| **Complex** | ***S. aureus* strains** | **MIC**  **(****µM)** | ***E. coli* strains** | **MIC**  **(µM)** | **References** |
| --- | --- | --- | --- | --- | --- |
| *N-heterocyclic carbene (NHC) silver(I) complexes* | | | |  |  |
| SBC3 | BH1CC | 22.0 | NCIB strain 9485 | 11.0 | 15 |
| Ag1 | ATCC 12,600 | 12.5 | ATCC 25,922 | 1.6 | 16 |
| Ag2 | ATCC 12,600 | 6.3 | ATCC 25,922 | 1.6 | 16 |
| *Bismuth(III) complexes* | | | |  |  |
| (CH_2_S)_2_BiCl | ATCC 33591 ATCC 43300 29,213 25,923 | 37.1  37.1  37.1  37.1 | ATCC 25922  MR *E. coli* 1042  ESBL *E. coli* 4036 | 37.1  37.1  37.1 | 17 |
| O(CH_2_CH_2_S)_2_BiCl | ATCC 33591 ATCC 43300 29,213  25,923 | 32.8  32.8  32.8  32.8 | ATCC 25922  MR *E. coli* 1042  ESBL E. coli 4036 | 32.8  32.8  32.8 | 17 |
| [BiL^1^Cl_2_] | ATCC 25923 | 7.0 | ATCC 25922  BH 100  MC 1061 | >212.0  53.0  >212.0 | 18 |
| [BiL^2^Cl_2_] | ATCC 25923 | 11.0 | ATCC 25922  BH 100  MC 1061 | 172.0  3.0  43.0 | 18 |
| *Ruthenium(II)-arene complexes* | | | |  |  |
| [4]PF_6_ | E40 | 175.3 | E72 | 175.3 | 19 |
| [(*p*-cym)Ru(O-cy-ind-th)Cl] | ATCC 29213 | 2.9 | ATCC 11775  I469 ESBL  J53 2138E | 94.5  94.5  47.25 | 20 |
| [(*p*-cym)Ru(O-ph-ind-th)Cl] | ATCC 29213 | 23.9 | ATCC 11775  I469 ESBL  J53 2138E | 191.2  191.2  191.2 | 20 |
| *Ruthenium(II)-polypyridyl complexes* | | | |  |  |
| [Ru(2,9-Me_2_phen)_2_dppz]^2+^ | MSSA160 MRSA41  MRSA252 | 10.0  5.0  2.5 | Not active on  *E. coli* MC4100 |  | 21 |
| X-03 | 29213 | 64.5 | K12 | >200.0 | 22 |
| [Ru(bpy)_2_(methionine)]^2+^ | ATCC 25923 | 73.3 | ATCC 11303 | 586.4 | 23 |
| [Ru(bpy)_2_curcumin]^+^ | ATCC 29,213 | 1.1 | ATCC 25,922 | >69.1 | 24 |
| [Ru(phen)_2_curcumin]^+^ | ATCC 29,213 | 1.0 | ATCC 25,922 | >65.7 | 24 |
| Mono-bb_7_ | MSSA ATCC 25,923  MRSA (JCU culture collection) | 4.5  17.8 | ATCC 25922 | 17.8 | 25 |
| *cis*-α-[Ru(phen)bb_12_]^2+^ | MSSA ATCC 25,923  MRSA (JCU culture collection) | 0.5  4.1 | ATCC 25922 | 8.3 | 26 |
| *cis*-β-[Ru(phen)(bb_12_)]^2+^ | MSSA ATCC 25,923  MRSA (JCU culture collection) | 0.5  4.1 | ATCC 25922 | 16.5 | 26 |
| [Ru(bb_7_)(dppz)]^2+^ | SH 1000  MRSA USA 300 LAC JE2 | 2.4  2.4 | MG1655 | 9.8 | 27 |
| [Ru(Me_4_phen)_2_(dppz)]^2+^ | SH1000 | 11.3 | MG1655  EC958 | 5.7  5.7 | 28 |
| [Ru(hexpytri)_3_](PF_6_)_2_ | MSSA ATCC 25,923  MSSA NZRM 9653  MRSA MR 9519 | 8.5  1.1  4.3 | Not active on *E. coli* ATCC 25,922 |  | 29 |
| [Ru(hexyltripy) (heptyltripy)]Cl_2_ | ATCC 25,923 | 2.0 | ATCC 25,922 | 17.0 | 30 |
| ΔΔ-Rubb_12_ | MSSA ATCC 25,923  MRSA (JCU culture collection) | 0.7  0.7 | ATCC 25922 | 1.4 | 31 |
| ΔΔ-Rubb_16_ | MSSA ATCC 25,923  MRSA (JCU culture collection) | 0.7  0.7 | ATCC 25922 | 2.6 | 31 |
| [Ru_2_(Me_4_phen)_2_(tpphz)]^4+^ | MSSA SH1000 | 40.0 | WT MG1655 EC958 ST131 (multi-drug-resistant, clinical isolate) | 1.2  1.6 | 32 |
| Cl-Rubb_12_-Cl | MSSA ATCC 25,923  MRSA (JCU culture collection) | 0.8  0.8 | ATCC 25,922 | 1.7 | 33 |
| Cl-Rubb_16_-Cl | MSSA ATCC 25,923 MRSA (JCU culture collection) | 6.4  6.4 | ATCC 25,922 | 6.4 | 33 |
| Rubb_7_-tetra (Rubb_7_-TL) | MSSA ATCC 25,923  MRSA (JCU culture collection) | 2.6  5.2 | MG1655 | 5.2 | 34 |
| Rubb_7_-TNL | MSSA ATCC 25,923  MRSA (JCU culture collection) | 1.3  2.6 | MG1655 | 2.6 | 34 |


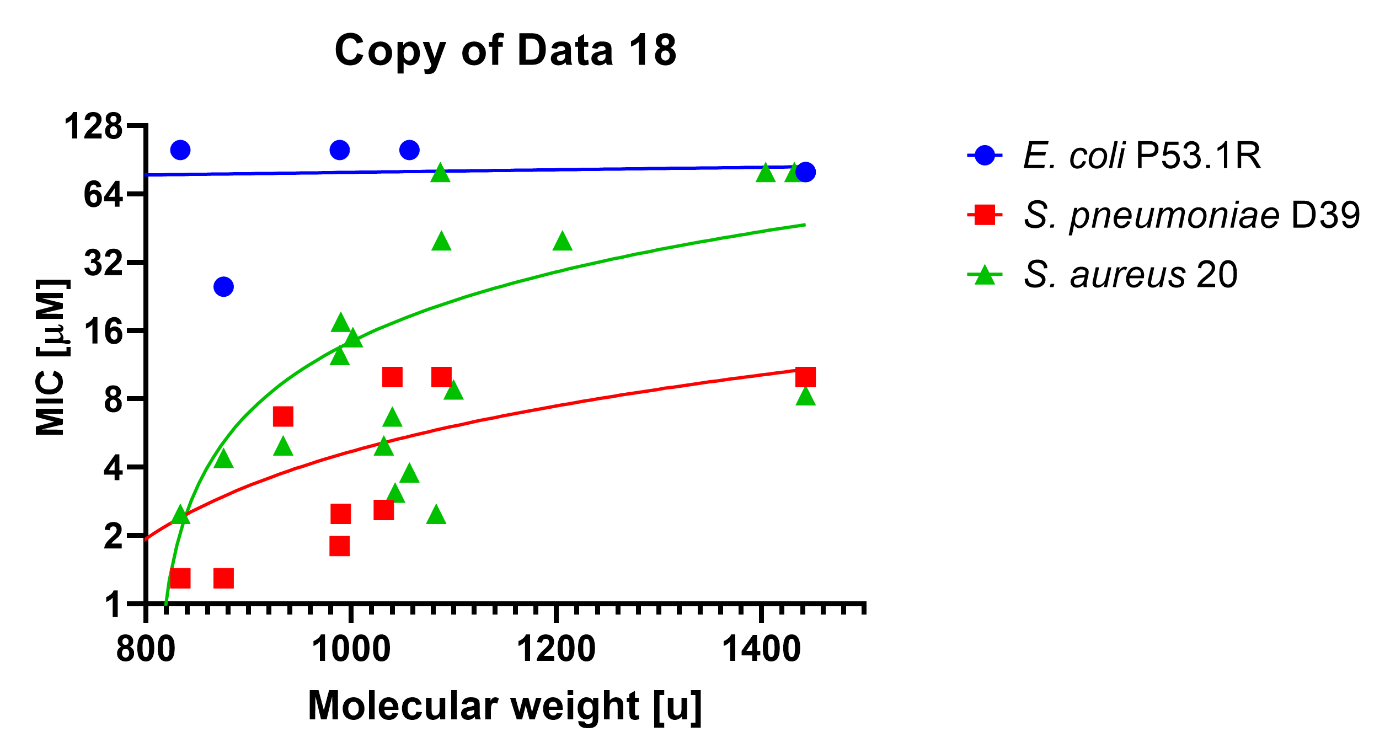


**Figure S10.** MIC values of the 22 diruthenium complexes included in this study as a function of their respective molecular weight. The linear regression (trendline) for each bacterial species is also shown.


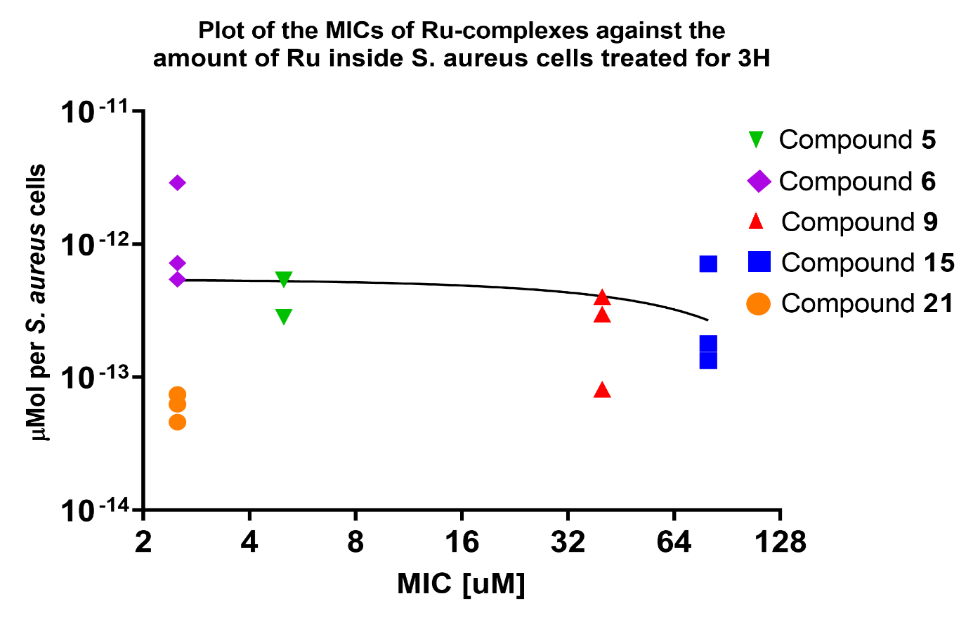

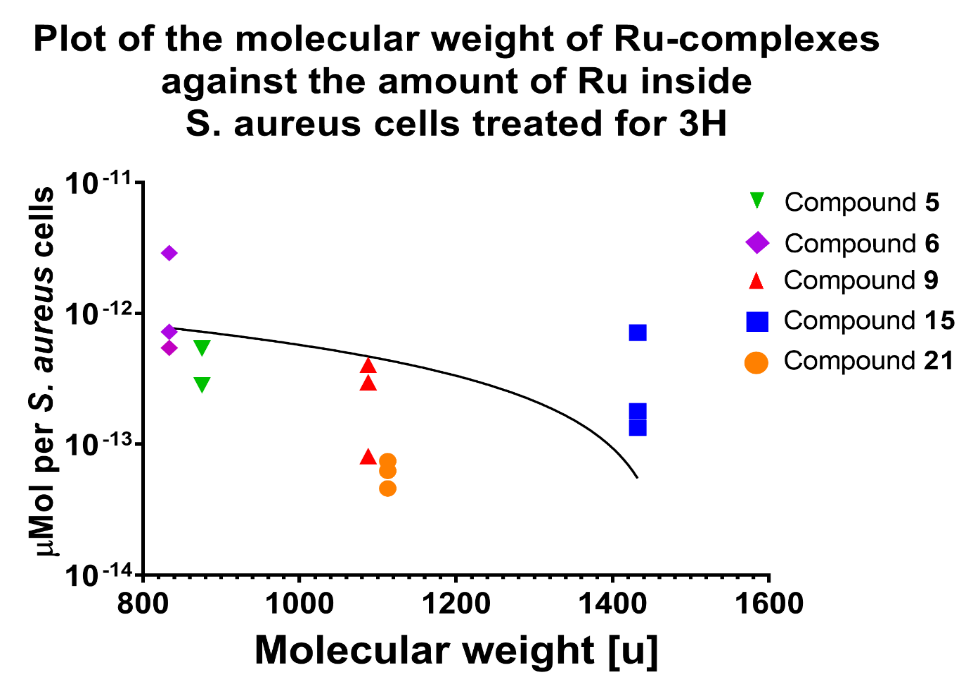


**Figure S11.** Top: Cellular uptake of compounds **5**, **6**, **9**, **15** and **21** as a function of the MIC values against S. aureus 20. Bottom: Cellular uptake of compounds **5**, **6**, **9**, **15** and **21** as a function of the molecular weight.


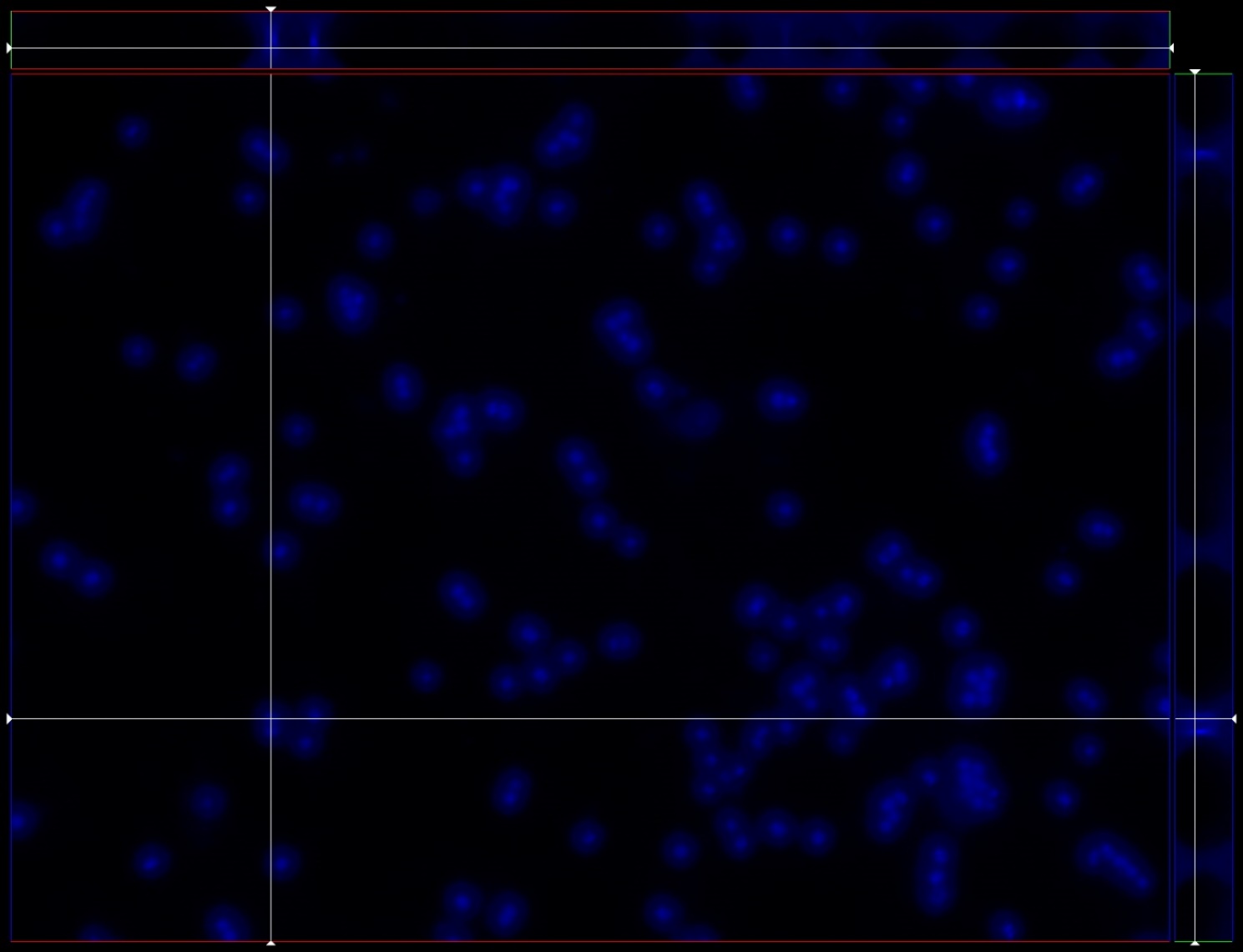


**Figure S12**. Z-cut image under DAPI filter (445/450 nm) of S. aureus 20 cells treated with compound **15** for 3 h. The Z-cut view is centred on a cell with fluorescence emission from both DAPI and compound **15**.


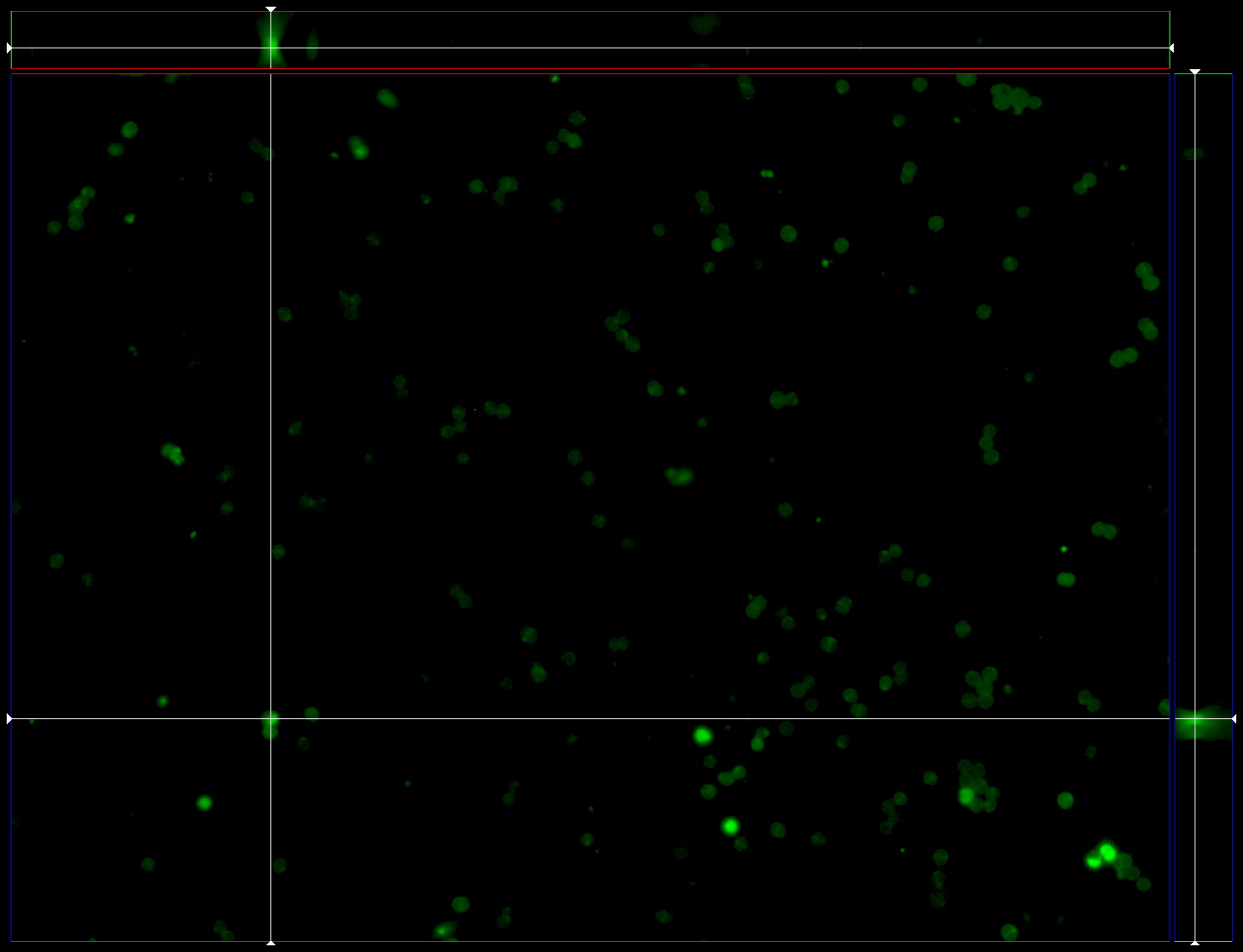


**Figure S13.** Z-cut image under GFP filter (525/550 nm) of *S. aureus* 20 cells treated with compound **15** for 3 h. The Z-cut view is centred on a cell with fluorescence emission from both DAPI and compound **15**.


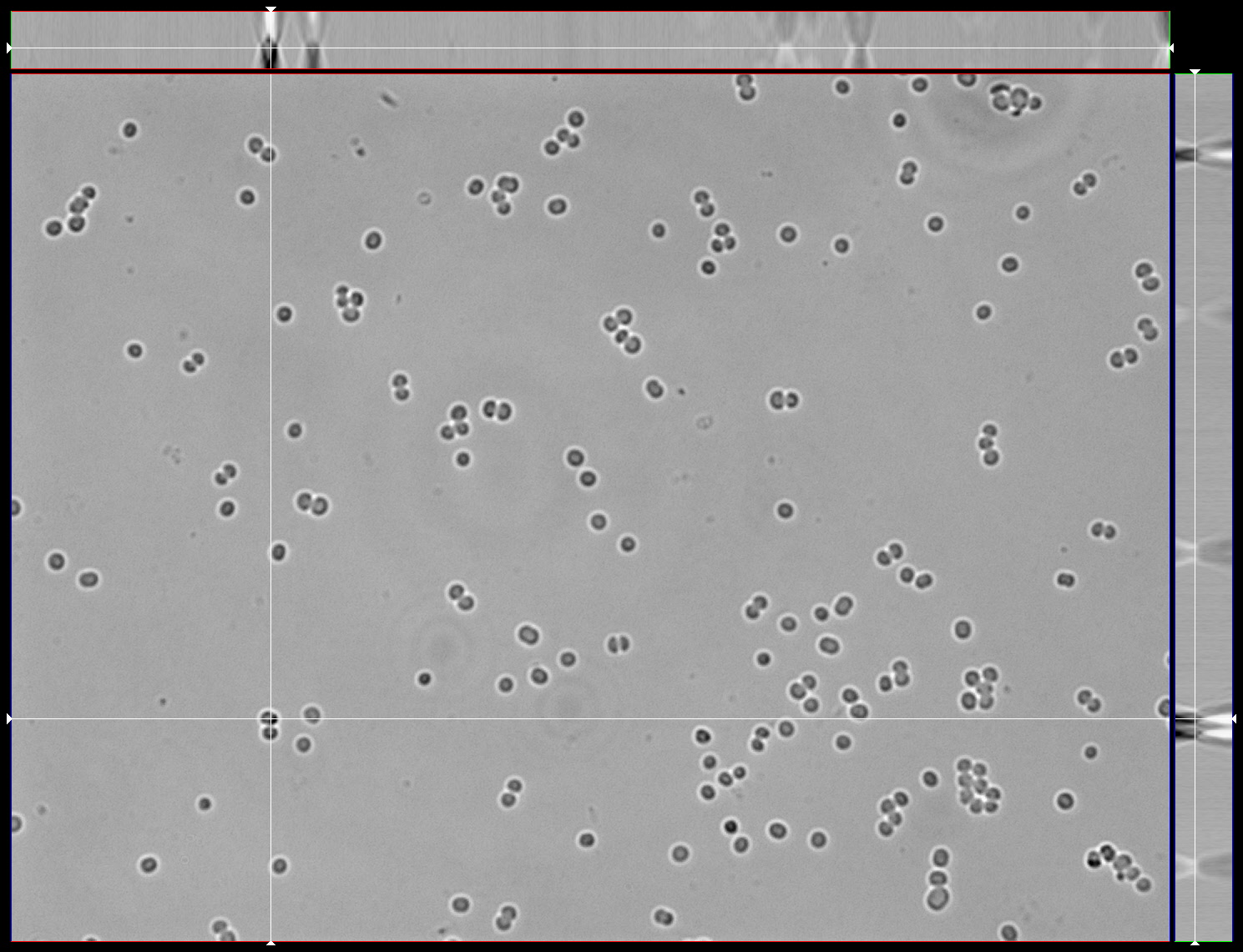


4.5 µm

**Figure S14.** Z-cut image under Brightfield of *S. aureus* 20 cells treated with compound **15** for 3 h. The Z-cut view is centred on a cell with fluorescence emission from both DAPI and compound **15**.


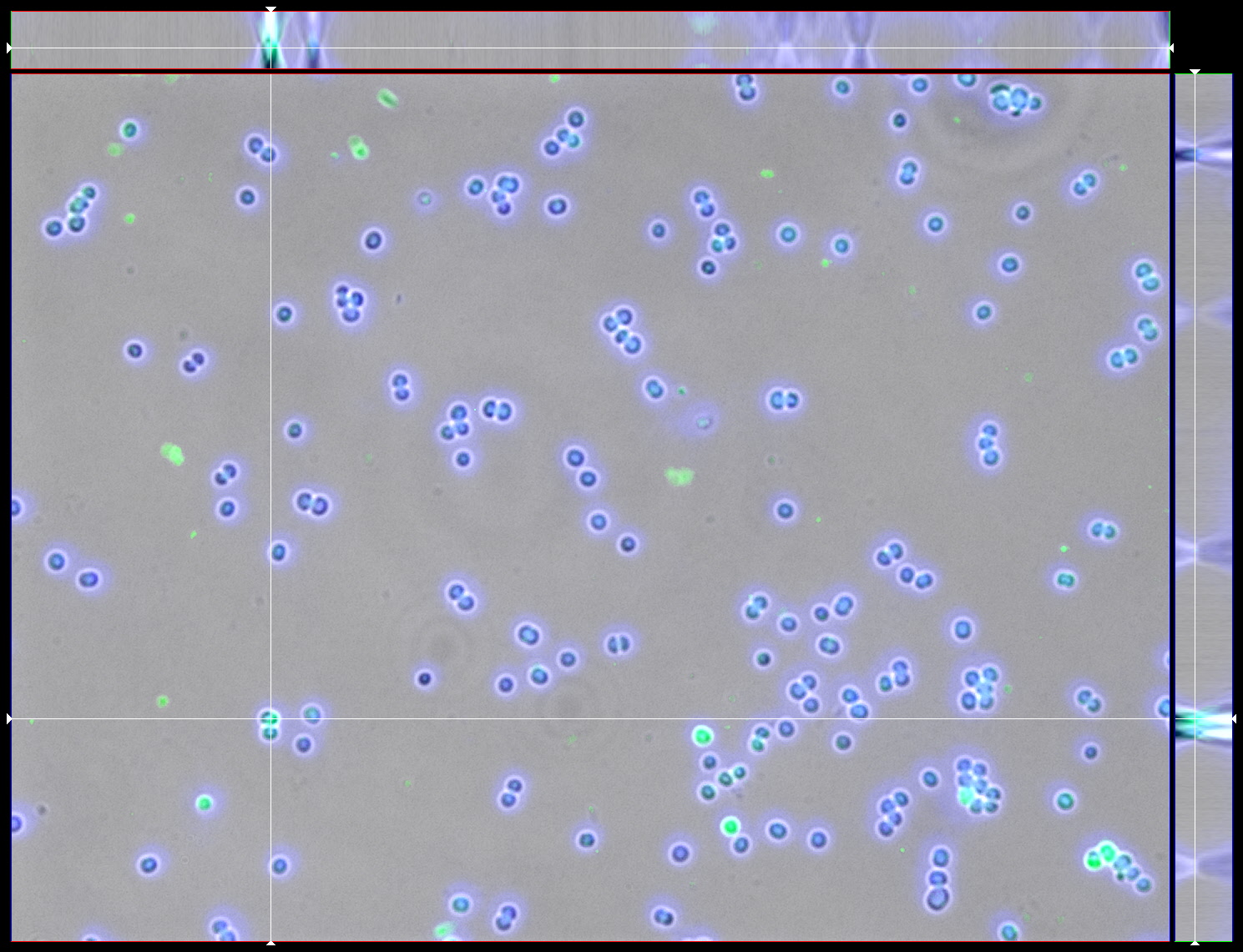


**Figure S15.** Merged Z-cut image with DAPI filter (445/450 nm), GFP filter (525/550 nm) and Brightfield of *S. aureus* 20 cells treated with compound **15** for 3 h. The Z-cut view is centred on a cell with fluorescence emission from both DAPI and compound **15**, highlighting their overlap inside of the cell.

**Table S2.** Effect of compounds **1-16** at 2.5 µM on HFF viability.

| Compound | HFF viability at 2.5 μM (%) |
| --- | --- |
| 1 | 56 |
| 2 | 51 |
| 3 | 99 |
| 4 | / |
| 5 | 86 |
| 6 | 99 |
| 7 | 56 |
| 8 | 72 |
| 9 | 0 |
| 10 | 0 |
| 11 | / |
| 12 | 97 |
| 13 | 93 |
| 14 | / |
| 15 | 95 |
| 16 | 97 |
| 17 | / |
| 18 | / |
| 19 | / |
| 20 | / |
| 21 | / |
| 22 | / |

**References**

(1) Giannini, F.; Paul, L. E. H.; Furrer, J.; Therrien, B.; Süss-Fink, G. Highly Cytotoxic Diruthenium Trithiolato Complexes of the Type [(Η6-p-MeC6H4Pri)2Ru2(Μ2-SR)3]+: Synthesis, Characterization, Molecular Structure and in Vitro Anticancer Activity. *New J. Chem.* **2013**, *37* (11), 3503–3511. https://doi.org/10.1039/C3NJ00476G.

(2) Păunescu, E.; Boubaker, G.; Desiatkina, O.; Anghel, N.; Amdouni, Y.; Hemphill, A.; Furrer, J. The Quest of the Best – A SAR Study of Trithiolato-Bridged Dinuclear Ruthenium(II)-Arene Compounds Presenting Antiparasitic Properties. *European Journal of Medicinal Chemistry* **2021**, *222*, 113610. https://doi.org/10.1016/j.ejmech.2021.113610.

(3) Ibao, A.-F.; Gras, M.; Therrien, B.; Süss-Fink, G.; Zava, O.; Dyson, P. J. Thiolato-Bridged Arene–Ruthenium Complexes: Synthesis, Molecular Structure, Reactivity, and Anticancer Activity of the Dinuclear Complexes [(Arene)2Ru2(SR)2Cl2]. *European Journal of Inorganic Chemistry* **2012**, *2012* (9), 1531–1535. https://doi.org/10.1002/ejic.201101057.

(4) Holzer, I.; Desiatkina, O.; Anghel, N.; Johns, S. K.; Boubaker, G.; Hemphill, A.; Furrer, J.; Păunescu, E. Synthesis and Antiparasitic Activity of New Trithiolato-Bridged Dinuclear Ruthenium(II)-Arene-Carbohydrate Conjugates. *Molecules* **2023**, *28* (2), 902. https://doi.org/10.3390/molecules28020902.

(5) Desiatkina, O.; Anghel, N.; Boubaker, G.; Amdouni, Y.; Hemphill, A.; Furrer, J.; Paunescu, E. Trithiolato-Bridged Dinuclear Ruthenium(II)-Arene Conjugates Tethered with Lipophilic Units: Synthesis and Antiparasitic Activity. ChemRxiv December 6, 2022. https://doi.org/10.26434/chemrxiv-2022-6ksq3. (*Journal of Organometallic Chemistry*, 2023, Accepted for publication.)

(6) Desiatkina, O.; Boubaker, G.; Anghel, N.; Amdouni, Y.; Hemphill, A.; Furrer, J.; Păunescu, E. Synthesis, Photophysical Properties and Biological Evaluation of New Conjugates BODIPY: Dinuclear Trithiolato-Bridged Ruthenium(II)-Arene Complexes. *ChemBioChem* **2022**, *23* (23), e202200536. https://doi.org/10.1002/cbic.202200536.

(7) Figuly, G. D.; Loop, C. K.; Martin, J. C. Directed Ortho-Lithiation of Lithium Thiophenolate. New Methodology for the Preparation of Ortho-Substituted Thiophenols and Related Compounds. *J. Am. Chem. Soc.* **1989**, *111* (2), 654–658. https://doi.org/10.1021/ja00184a038.

(8) Thomas, A. A.; Zahrt, A. F.; Delaney, C. P.; Denmark, S. E. Elucidating the Role of the Boronic Esters in the Suzuki–Miyaura Reaction: Structural, Kinetic, and Computational Investigations. *J. Am. Chem. Soc.* **2018**, *140* (12), 4401–4416. https://doi.org/10.1021/jacs.8b00400.

(9) Someya, H.; Itoh, T.; Aoki, S. Synthesis of Disaccharide Nucleosides Utilizing the Temporary Protection of the 2′,3′-Cis-Diol of Ribonucleosides by a Boronic Ester. *Molecules* **2017**, *22* (10), 1650. https://doi.org/10.3390/molecules22101650.

(10) Niu, Q.; Xu, X.; Sun, H.; Li, X. Synthesis of 2-Mercaptobenzaldehyde, 2-Mercaptocyclohex-1-Enecarboxaldehydes and 3-Mercaptoacrylaldehydes. *Chinese Journal of Chemistry* **2012**, *30* (10), 2495–2500. https://doi.org/10.1002/cjoc.201200433.

(11) Zampieri, D.; Vio, L.; Fermeglia, M.; Pricl, S.; Wünsch, B.; Schepmann, D.; Romano, M.; Mamolo, M. G.; Laurini, E. Computer-Assisted Design, Synthesis, Binding and Cytotoxicity Assessments of New 1-(4-(Aryl(Methyl)Amino)Butyl)-Heterocyclic Sigma 1 Ligands. *European Journal of Medicinal Chemistry* **2016**, *121*, 712–726. https://doi.org/10.1016/j.ejmech.2016.06.001.

(12) Igoe, N.; Bayle, E. D.; Tallant, C.; Fedorov, O.; Meier, J. C.; Savitsky, P.; Rogers, C.; Morias, Y.; Scholze, S.; Boyd, H.; Cunoosamy, D.; Andrews, D. M.; Cheasty, A.; Brennan, P. E.; Müller, S.; Knapp, S.; Fish, P. V. Design of a Chemical Probe for the Bromodomain and Plant Homeodomain Finger-Containing (BRPF) Family of Proteins. *J. Med. Chem.* **2017**, *60* (16), 6998–7011. https://doi.org/10.1021/acs.jmedchem.7b00611.

(13) Korin, E.; Cohen, B.; Liu, Y.-D.; Zeng, C.-C.; Shames, A. I.; Becker, J. Y. Examining the Binding Mechanism of 3,4-Dihydro-3-(2-Oxo-2-Phenylethylidene)-Quinoxalin-2(1H)-One and Its Fragments to Cu2+. *Journal of Coordination Chemistry* **2013**, *66* (13), 2351–2366. https://doi.org/10.1080/00958972.2013.803535.

(14) Dorogov, M. V.; Ivanovsky, S. A.; Khakhina, M. Y.; Kravchenko, D. V.; Tkachenko, S. E.; Ivachtchenko, A. V. Synthesis of 7‐Sulfamoyl‐substituted 2‐Oxo‐2,3,4,5‐tetrahydro‐1H‐benzo[b]Azepines. *Synthetic Communications* **2006**, *36* (23), 3525–3535. https://doi.org/10.1080/00397910600943493.

(15) Bellale, E. V.; Chaudhari, M. K.; Akamanchi, K. G. A Simple, Fast and Chemoselective Method for the Preparation of Arylthiols. *Synthesis* **2009**, 3211–3213. https://doi.org/10.1055/s-0029-1216955.
